# Maternal and perinatal outcomes following a diagnosis of Hodgkin lymphoma during or prior to pregnancy: A systematic review

**DOI:** 10.1111/1471-0528.17347

**Published:** 2022-12-12

**Authors:** Orla A. Houlihan, Daire Buckley, Gillian M. Maher, Fergus P. McCarthy, Ali S. Khashan

**Affiliations:** ^1^ School of Public Health University College Cork Cork Ireland; ^2^ INFANT Research Centre University College Cork Cork Ireland; ^3^ Department of Obstetrics and Gynaecology University College Cork Cork Ireland

**Keywords:** Hodgkin lymphoma, maternal, perinatal, pregnancy

## Abstract

**Background:**

The initial peak incidence of Hodgkin lymphoma (HL) occurs during reproductive years.

**Objectives:**

Synthesise published literature on the relationship between HL and maternal and perinatal outcomes.

**Search strategy:**

Systematic search of PubMed/Medline, Cochrane Library, Scopus, Embase and Science Direct from inception to June 2022, supplemented by hand‐searching reference lists.

**Selection criteria:**

Two reviewers independently reviewed titles, abstracts and full‐text articles. Published studies containing original data were eligible.

**Data Collection and Analysis:**

Two reviewers independently extracted data and appraised study quality. Outcomes for pregnant women with a previous/current diagnosis of HL were compared separately with women never diagnosed with HL. Where data permitted, meta‐analyses of odds ratios and proportions were performed. Certainty of evidence was determined using the Grading of Recommendations Assessment, Development and Evaluation (GRADE) framework.

**Main results:**

Of the 5527 studies identified, 33 met the inclusion criteria. In the groups with HL before pregnancy and HL during pregnancy, adjusted odds ratios were not statistically significant for congenital malformation (aOR 1.7, 95% CI 0.9–3.1, and aOR 1.84, 95% CI 0.81–4.15, respectively), preterm birth (PTB) (aOR 0.99, 95% CI 0.65–1.51, and aOR 6.74, 95% CI 0.52–88.03, respectively) and miscarriage (aOR 0.78, 95% CI 0.55–1.10, and aOR 0.38, 95% CI 0.05–2.72, respectively). The aORs for all other outcomes were not statistically significant, except for blood transfusion (aOR 1.38, 95% CI 1.05–1.82) and venous thromboembolism (VTE) (aOR 7.93, 95% CI 2.97–21.22) in the group for HL during pregnancy. The proportion of anaemia was also increased in this group (69%, 95% CI 57%–80% vs 4%, 95% CI 4%–5%, respectively). The GRADE certainty of findings ranged from low to very low.

**Conclusions:**

Rates of most adverse pregnancy outcomes among women with a previous/current HL diagnosis are not increased significantly compared with the general pregnant population. Women with HL diagnosed during pregnancy may have a higher PTB rate and increased likelihood of VTE, anaemia and blood transfusion; however, small study numbers and the low to very low GRADE certainty of findings preclude firm conclusions.

## 1 INTRODUCTION

Hodgkin lymphoma (HL) is a haematological malignancy that can affect adolescents, young adults and women of childbearing age.^1^ Prognosis has improved steadily over recent decades, with a 5‐year overall survival of >95% reported for female patients of childbearing age.^2^ Up to 3% of patients are diagnosed during pregnancy.^3^ Although reduced ovarian function has been reported, it recovers after treatment for HL for most female patients <35 years of age.^4^

The treatment of HL usually involves chemotherapy and radiotherapy, which can increase the risk of adverse pregnancy outcomes.^5^ Chemotherapy is cytotoxic in nature and can interfere with cell metabolism and cell‐cycle progression, causing congenital malformations as well as placental insufficiency and intrauterine growth restriction (IUGR).^6^, ^7^ Radiotherapy restricts uterine blood flow and growth and can cause teratogenic effects through the induction of DNA damage and mutations.^8^, ^9^

Women diagnosed with HL during pregnancy are usually advised to avoid chemotherapy during the first trimester as this is a critical time for fetal organogenesis.^6^ When administered during the second and third trimesters of pregnancy, chemotherapy can still be associated with adverse outcomes as a result of fetal growth restriction, including preterm birth (PTB) and low birthweight (LBW).^6^, ^10^ Typically, radiotherapy treatment for HL is delivered after the completion of chemotherapy.^11^ As associated risks to the fetus include PTB, LBW and small for gestational age (SGA), caused by uterine blood flow restriction, radiotherapy treatment is usually deferred until after delivery.^12^ Previous pelvic or abdominal radiotherapy can result in a smaller uterine volume and reduced uterine distensibility.^9^, ^13^, ^14^ This can restrict fetal growth, increasing the risk of miscarriage,^15^ PTB,^16^, ^17^, ^18^, ^19^ caesarean section (CS),^17^ LBW,^13^, ^17^, ^19^ low 5‐min APGAR score,^20^ and postpartum haemorrhage (PPH).^18^

Previous systematic reviews of pregnant women with a history of cancer report increased risks of adverse maternal and perinatal outcomes, including LBW, PPH, and PTB, compared with controls; however, none of these outcomes are specific to HL.^21^, ^22^ Systematic reviews specific to lymphoma during pregnancy are lacking; however, reviews of other haematological malignancies active during pregnancy, including acute promyelocytic leukaemia and acute myeloid leukaemia,^23^, ^24^ report adverse outcomes, including PTB, miscarriage and elective termination.

Given the potential adverse pregnancy outcomes related to malignancy, chemotherapy and radiotherapy, a systematic review was undertaken with the aim of providing a comprehensive summary of the published literature regarding maternal and perinatal outcomes following a diagnosis of HL prior to or during pregnancy.

## 2 METHODS

### 2.1 Search methodology

A systematic review was performed in accordance with a pre‐prepared protocol approved by the Social Research Ethics Committee of the School of Public Health at University College Cork following the Meta‐analysis of Observational Studies in Epidemiology (MOOSE) guidelines, described in Appendix S1. PubMed/Medline, the Cochrane Library, Scopus, Embase and Science Direct were systematically searched by OH from inception to 6 February 2022. Full search terms are described in Appendix S2. There were no restrictions on language, location of study or publication date. Searches of the electronic databases were supplemented by hand‐searching the reference lists of included studies for further potentially eligible studies. Results from each database were imported into the reference management software EndNote™ 20 (Clarivate, Philadelphia, PA, USA). Where full text was not available by search, corresponding authors were contacted directly by email. Two reviewers (OH and DB) independently screened the titles and abstracts of retrieved studies and excluded studies that clearly did not meet the inclusion criteria. Full‐text articles for the remaining studies were obtained and assessed for eligibility. If consensus could not be achieved, disagreements were resolved following the involvement of a third reviewer (AK). A repeat search of all databases was performed prior to the final analysis on 15 June 2022 to identify any additional eligible studies published since the initial search.

### 2.2 Study inclusion and exclusion criteria

The population of interest was pregnant women and their children, exposure was a previous or current diagnosis of any histological subtype of HL and the comparator was no previous or current diagnosis of HL. Primary outcomes were congenital malformation, PTB and miscarriage. Secondary outcomes were: prelabour rupture of membranes (PROM); PPH; anaemia; 5‐min Apgar score of <7; LBW, defined as <2500 g; SGA, defined as birthweight less than the tenth centile for gestational age; neonatal death; pregnancy‐induced hypertension (PIH); pre‐eclampsia; gestational diabetes; elective termination of pregnancy; stillbirth; CS; induction of labour (IOL); blood transfusion; chorioamnionitis; and venous thromboembolism (VTE). Randomised controlled trials, cohort, case–control, cross‐sectional studies and case series containing original data were eligible for inclusion. Conference abstracts, clinical vignettes and letters to the editor were eligible provided raw data pertaining to maternal and/or perinatal outcomes were published. Excluded studies were case studies, review articles and commentaries. In cases where multiple publications using the same data existed, the most recent study was included, provided additional information was not included in earlier publications.

### 2.3 Data extraction

Data from eligible studies were manually extracted independently by two reviewers (OH and DB) into a predefined Microsoft® Excel® data collection spreadsheet (Microsoft, Redmond, WA, USA). Study title, author(s), country, journal, year of publication, study period, type of study, data sources, sample size, timing of HL diagnosis, timing of treatment received, assessment methods for outcomes and outcomes were recorded. Any discrepancies were resolved following discussion with a third reviewer (AK). Corresponding authors were contacted directly by email for any missing data or if additional data to those published were required. Data from studies not published in the English language were extracted initially with the aid of Google Translate and then verified by researchers fluent in the published language.

### 2.4 Risk‐of‐bias (quality) assessment

The quality of included studies was assessed independently by two reviewers (OH and DB) using the quality assessment tool described by McDonald et al.^25^ Any disagreements were discussed with a third reviewer (AK) to achieve consensus. The Grading of Recommendations, Assessment, Development and Evaluations (GRADE) framework was used to evaluate the certainty of results from meta‐analyses.^26^

### 2.5 Statistical analysis

Where sufficient data were present, meta‐analyses of proportions using a random‐effects model were performed using Stata® 17 (StataCorp LLC, College Station, TX, USA).^27^ The random‐effects model was used because of the heterogeneity among studies, arising from differences in study design, measurements and outcomes. Data were analysed according to three population groups: pregnant women diagnosed and treated for HL before pregnancy; women with a diagnosis of HL during pregnancy; and pregnant women never diagnosed with HL. All women diagnosed with HL during pregnancy were included in the main analysis rather than only women who received treatment to maximise the number of women included.

The ‘metaprop’ command in Stata® 17 was used to pool the proportions of outcomes and estimate the 95% confidence intervals (95% CIs).^28^ This command was also used to conduct separate analyses for studies that reported data separately according to the timing of therapy. The Freeman–Tukey double‐arcsine transformation was used to stabilise the variance in proportions of each study and to avoid the exclusion of studies with 0% prevalence.^28^ Separate meta‐analyses were performed using RevMan 5.4 (Cochrane, cochrane.org) for studies in which odds ratios (ORs) were provided or could be calculated from the data reported. Relative risks (RRs) were reported for a minority of studies and included separately in the results section. For outcomes where data were insufficient for meta‐analyses to be performed, results were reported by narrative synthesis.

The *I* ^2^ statistic was used to assess heterogeneity in meta‐analyses.^29^ Heterogeneity was explored by subgroup analyses according to study design, World Health Organization (WHO) region and risk of bias (low, low–moderate, moderate or high). Sensitivity analyses were performed in cases of heterogeneous outcome data reported by studies, for example, studies that only reported data on IUGR rather than SGA, and for data reported in a form other than a full article, to assess whether their exclusion influenced the results of the original meta‐analyses. A sensitivity analysis was also performed that excluded studies that did not specify gestation or trimester at diagnosis, as pregnant women diagnosed with HL after 24 weeks of gestation or in the third trimester are no longer at risk of miscarriage.^30^ As antenatal and cancer care have improved in recent years,^31^, ^32^ which may have independently influenced the incidence of adverse maternal and perinatal outcomes, two separate post hoc sensitivity analyses were performed excluding studies published before 2000 and before 1990. There were insufficient studies reporting OR or RR (three or fewer per outcome estimate) to test for funnel‐plot asymmetry and publication bias.^33^

## 3 RESULTS

### 3.1 Search results

The initial search produced 5527 results, after the removal of duplicates. After titles and abstracts were screened, 76 full‐text articles were reviewed. Authors of five articles,^34^, ^35^, ^36^, ^37^, ^38^ and three conference abstracts,^39^, ^40^, ^41^ were contacted by email for data specific to HL. Two responses were received; one provided the requested data.^38^ Five additional eligible articles were identified following a manual search of the reference lists,^12^, ^42^, ^43^, ^44^, ^45^ resulting in a total of 31 full articles,^12^, ^38^, ^42^, ^43^, ^44^, ^45^, ^46^, ^47^, ^48^, ^49^, ^50^, ^51^, ^52^, ^53^, ^54^, ^55^, ^56^, ^57^, ^58^, ^59^, ^60^, ^61^, ^62^, ^63^, ^64^, ^65^, ^66^, ^67^, ^68^, ^69^, ^70^ one vignette,^71^ and one letter to the editor included in the final systematic review (Appendix S3).^72^ Included studies described pregnancies of 3058 survivors of HL, 1150 women with HL and 25 196 997 women never diagnosed with HL. Two studies included that were not published in English were translated by Polish and German researchers who were native speakers known to the study authors.^49^, ^68^ The characteristics of the included studies are summarised in Tables S1 and S2.

### 3.2 Meta‐analysis results: primary outcomes

#### 3.2.1 Congenital malformations

**FIGURE 1:**
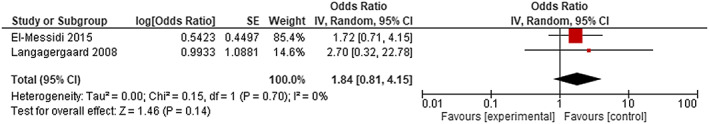
Meta‐analysis forest plot (random effects) of adjusted odds ratios for congenital malformations among studies of women with a diagnosis of HL during pregnancy

Twenty studies included congenital malformations as an outcome.^44^, ^46^, ^48^, ^49^, ^50^, ^51^, ^52^, ^53^, ^54^, ^57^, ^58^, ^61^, ^62^, ^63^, ^64^, ^65^, ^68^, ^69^, ^70^, ^72^ The adjusted ORs (aORs) for both the group treated for HL before pregnancy (one study, 11 events, 181 births, aOR 1.7, 95% CI 0.9–3.1, *I* ^2^ = not calculable, NC) and the group diagnosed with HL during pregnancy (two studies, six events, 651 births, aOR 1.84, 95% CI 0.81–4.15, *I* ^2^ = 0) were not statistically significant compared with pregnant women never diagnosed with HL (Figure 1; Table 1). The proportions of congenital malformations were 50 per 1000 births (95% CI 20–18 per 1000 births, *I* ^2^ = 58.86%, *p* = 0.01) among women treated for HL before pregnancy (10 studies, 38 events, 675 births),^46^, ^49^, ^50^, ^51^, ^57^, ^61^, ^62^, ^68^, ^69^, ^72^ and 10 per 1000 births (95% CI 0–40 per 1000 births, *I* ^2^ = 57.91%, *p* = 0.01) among women diagnosed with HL during pregnancy (12 studies, 14 events, 883 births).^44^, ^48^, ^52^, ^53^, ^54^, ^58^, ^61^, ^63^, ^64^, ^65^, ^70^, ^72^ These were not significantly different compared with the proportion of congenital malformations among women never diagnosed with HL (four studies, 31 846 events, 7 925 914 births, 30 per 1000 births, 95% CI 0–70 per 1000 births, *I* ^2^ = 99.57%, *p* = 0.00)^53^, ^57^, ^61^, ^63^ (Figure S1). Pooled proportions did not statistically significantly differ among those who received antenatal therapy (six studies, seven events, 166 births, 20 per 1000 births, 95% CI 0–100 per 1000 births, *I* ^2^ = 66.62%, *p* = 0.01) and among those who did not (three studies, one event, 54 births, 0 per 1000 births, 95% CI 0–10 per 1000 births, *I* ^2^ = NC, *p* = NC) (Figure S2).

**TABLE 1:** Crude and adjusted estimates of outcomes

	Number of studies (crude)	Number of events (crude)	Number of pregnancies/births (crude)	Crude OR (95% CI, *I* ^2^)	Number of studies (adjusted)	Number of events (adjusted)	Number of pregnancies /births (adjusted)	aOR (95% CI, *I* ^2^)
Congenital malformations								
Treatment for HL before pregnancy	1	8	84	0.74 (0.28–1.78, NC)	1	11	181	1.7 (0.9–3.1, NC)
HL diagnosis during pregnancy	1	5	638	1.98 (0.82–4.77, NC)	2	6	651	1.84 (0.81–4.15, 0)
Preterm birth								
Treatment for HL before pregnancy	2	67	419	1.66 (1.21–2.28, 0)	2	38	537	0.99 (0.65–1.51, 0)
HL diagnosis during pregnancy	3	100	759	2.02 (1.63–2.50, 0)	2	94	653	6.74 (0.52–88.03, 95)
Miscarriage								
Treatment for HL before pregnancy	1	7	93	0.85 (0.29–2.18, NC)	2	62	491	0.78 (0.55–1.10, 0)
HL diagnosis during pregnancy	1	1	638	0.38 (0.05–2.67, NC)	1	1	638	0.38 (0.05–2.72, NC)
Postpartum haemorrhage								
HL diagnosis during pregnancy	1	17	638	1.24 (0.76–2.00, NC)	1	17	638	1.25 (0.77–2.02, NC)
Anaemia								
Treatment for HL before pregnancy	–	–	–	–	1	4	61	1.0 (0.3–2.7, NC)
5‐min APGAR score of <7								
HL diagnosis during pregnancy	1	8	93	2.20 (0.92–4.53, NC)	–	–	–	–
Low birthweight								
Treatment for HL before pregnancy	1	20	337	1.44 (0.84–2.46, NC)	2	15	523	0.89 (0.43–1.82, 0)
HL diagnosis during pregnancy	1	9	94	2.23 (0.99–4.45, NC)	–	–	–	–
Small for gestational age								
Treatment for HL before pregnancy	1	30	337	0.97 (0.63–1.49, NC)	–	–	–	–
HL diagnosis during pregnancy	2	26	726	1.28 (0.86–1.90, 0)	1	15	638	1.17 (0.70–1.95, NC)
Neonatal death								
Treatment for HL before pregnancy	1	2	15	2.5 (0.3–9.0, NC)	–	–	–	–
Pregnancy‐induced hypertension								
HL diagnosis during pregnancy	1	14	638	0.70 (0.41–1.18, NC)	1	14	638	0.66 (0.39–1.12, NC)
Pre‐eclampsia								
Treatment for HL before pregnancy	–	–	–	–	1	4	61	0.7 (0.2–1.8, NC)
HL diagnosis during pregnancy	2	40	732	1.33 (0.97–1.83, 0)	1	33	638	1.3 (0.92–1.85, NC)
Gestational diabetes								
HL diagnosis during pregnancy	2	48	732	1.24 (0.92–1.66, 0)	1	43	638	1.28 (0.94–1.75, NC)
Elective termination								
Treatment for HL before pregnancy	–	–	–	–	1	43	346	1.1 (0.7–1.8, NC)
Stillbirth								
Treatment for HL before pregnancy	1	2	82	1.70 (0.14–15.10, NC)	2	4	538	1.59 (0.49–5.17, 0)
Caesarean section								
HL diagnosis during pregnancy	2	224	663	0.92 (0.48–1.75, 0)	1	219	638	1.14 (0.97–1.34, NC)
Induction of labour								
HL diagnosis during pregnancy	1	10	638	1.28 (0.69–2.40, NC)	1	10	638	1.28 (0.68–2.38, NC)
Blood transfusion								
HL diagnosis during pregnancy	1	55	638	1.49 (1.13–1.96, NC)	1	55	638	1.38 (1.05–1.82, NC)
Chorioamnionitis								
HL diagnosis during pregnancy	1	10	638	0.90 (0.48–1.68, NC)	1	10	638	0.91 (0.48–1.69, NC)
Venous thromboembolism								
HL diagnosis during pregnancy	1	4	638	8.74 (3.27–23.36, NC)	1	4	638	7.93 (2.97–21.22, NC)

#### 3.2.2 Preterm birth

**FIGURE 2:**
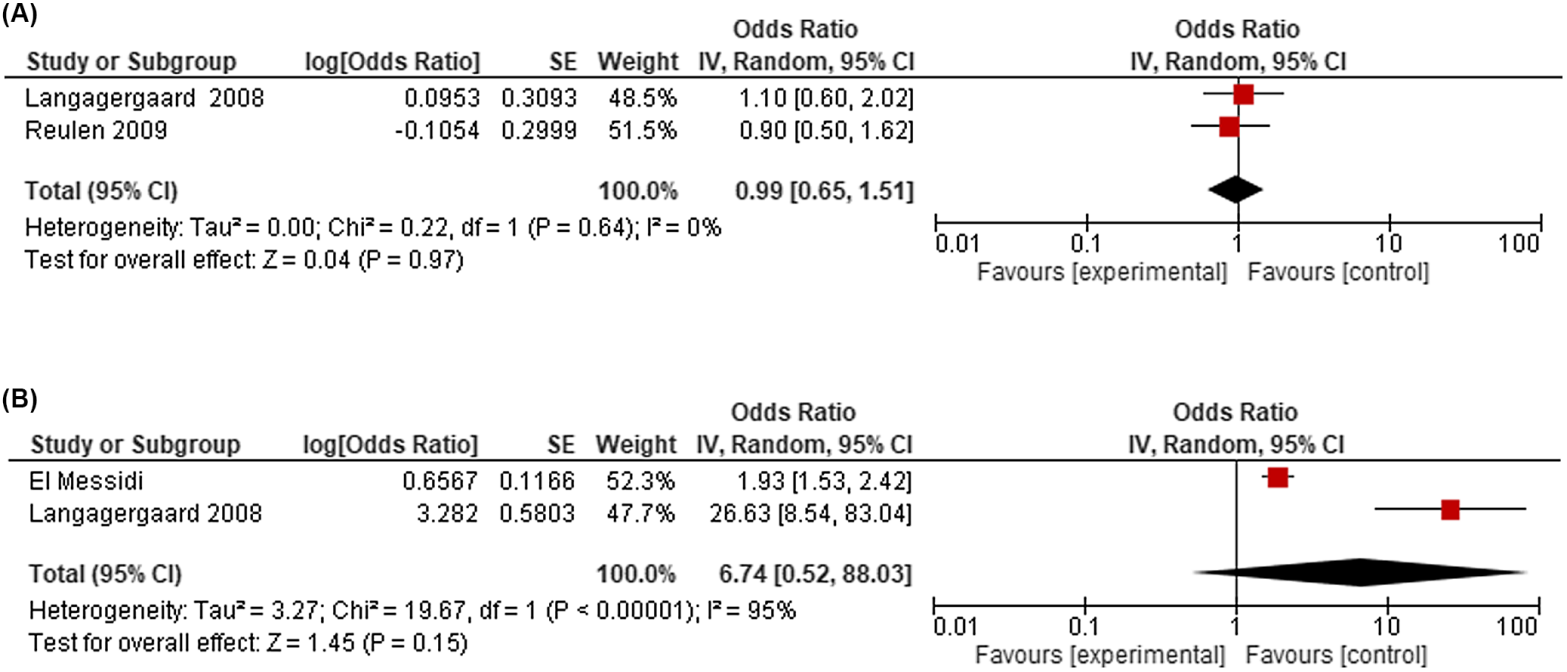
(a) Meta‐analysis forest plot (random effects) of adjusted odds ratios for preterm birth among studies of women treated for HL prior to pregnancy. (b) Meta‐analysis forest plot (random effects) of adjusted odds ratios for preterm birth among studies of women with a diagnosis of HL during pregnancy

There were 21 studies in the PTB meta‐analysis.^12^, ^43^, ^46^, ^47^, ^48^, ^51^, ^53^, ^54^, ^56^, ^57^, ^61^, ^63^, ^64^, ^65^, ^66^, ^68^, ^69^, ^70^, ^71^, ^72^, ^73^ Only three studies specified whether PTB was planned or spontaneous.^54^, ^61^, ^64^ Pooled aORs were not statistically significant for both the group treated for HL before pregnancy, which contained 38 events among two studies of 537 births (0.99, 95% CI 0.65–1.51, *I* ^2^ = 0%), and the group diagnosed with HL during pregnancy, which contained 94 events among two studies of 653 births (6.74, 95% CI 0.52–88.03, *I* ^2^ = 95%), compared with pregnant women never diagnosed with HL (Figure 2; Table 1). In the group diagnosed with HL during pregnancy (12 studies, 189 events among 1036 births),^48^, ^53^, ^54^, ^61^, ^63^, ^64^, ^65^, ^66^, ^70^, ^71^, ^72^, ^73^ the proportion of PTB was higher (18%, 95% CI 10–28%, *I* ^2^ = 86.35%, *p* = 0.00) than in the group with HL before pregnancy (10 studies, 176 events among 1697 births, 8%, 95% CI 5–12%, *I* ^2^ = 82.97%, *p* = 0.00),^12^, ^43^, ^46^, ^47^, ^51^, ^56^, ^57^, ^61^, ^68^, ^69^ and the group never diagnosed with HL (nine studies, 995 084 events among 12 377 598 births, 7%, 95% CI 6–8%, *I* ^2^ = 99.96%, *p* = 0.00) (Figure S3).^12^, ^47^, ^53^, ^56^, ^57^, ^61^, ^63^, ^69^, ^73^ There was no significant difference in proportions among those who received antenatal therapy (six studies, 47 events, 176 births, 19%, 95% CI 6–36%, *I* ^2^ = 81.21%, *p* = 0.00) compared with those who did not (two studies, 26 events, 55 births, 47%, 95% CI 34–61%, *I* ^2^ = NC, *p* = NC) (Figure S4).

#### 3.2.3 Miscarriage

**FIGURE 3:**
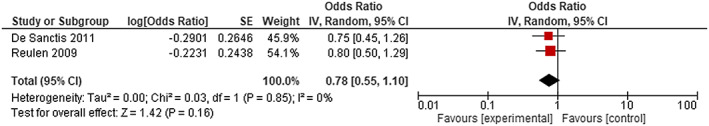
Meta‐analysis forest plot (random effects) of adjusted odds ratios for miscarriage among studies of women treated for HL prior to pregnancy

Twenty‐three studies included miscarriage as an outcome.^43^, ^44^, ^46^, ^48^, ^49^, ^50^, ^51^, ^52^, ^53^, ^54^, ^55^, ^57^, ^58^, ^60^, ^62^, ^63^, ^64^, ^66^, ^68^, ^69^, ^70^, ^71^, ^72^ Pooled aORs for miscarriage were also not significant for either the group treated for HL before pregnancy (two studies, 62 events, 491 pregnancies, aOR 0.78, 95% CI 0.55–1.10, *I* ^2^ = 0%) or the group diagnosed with HL during pregnancy (one study, one event, 638 pregnancies, aOR 0.38, 95% CI 0.05–2.72, *I* ^2^ = NC), compared with pregnant women never diagnosed with HL (Figure 3; Table 1).^53^ Compared with the group never diagnosed with HL (four studies, 33 266 events, 7 918 650 pregnancies, 7%, 95% CI 0–22%, *I* ^2^ = 99.67%, *p* = 0.00), the proportion of miscarriages was not significantly increased in the group treated for HL before pregnancy (12 studies, 266 events, 2024 pregnancies, 10%, 95% CI 7–13%, *I* ^2^ = 60.72%, *p* = 0.00),^43^, ^46^, ^49^, ^50^, ^51^, ^55^, ^57^, ^60^, ^62^, ^68^, ^69^, ^72^ or the group diagnosed with HL during pregnancy (13 studies, 14 events, 1009 pregnancies, 1%, 95% CI 0–5%, *I* ^2^ = 68.29%, *p* = 0.00) (Figure S5).^44^, ^48^, ^49^, ^52^, ^53^, ^54^, ^58^, ^63^, ^64^, ^66^, ^70^, ^71^, ^72^ There was no difference according to the timing of therapy in the group with HL diagnosed during pregnancy, with pooled proportions of 0% for both women who did (seven studies, three events, 114 pregnancies) and women who did not (three studies, zero events, 20 pregnancies) receive antenatal therapy (Figure S6).

### 3.3 Meta‐analysis results: secondary outcomes

The meta‐analysis results of secondary outcomes are summarised in Table 1 and Figures S7–S24. Proportions of PROM (17 events, 11%, 95% CI 7–17%, *I* ^2^ = NC, *p* = NC) and PPH (18 events, 12%, 95% CI 8–18%, *I* ^2^ = NC, *p* = NC) among women with a history of HL were higher in one cohort study, of 153 births,^42^ than the pooled proportions of PROM (1939 events, 0%, 95% CI 0–0%, *I* ^2^ = NC, *p* = NC) (Figure S7) and PPH (173 830 events, 2%, 95% CI 2–2%, *I* ^2^ = NC, *p* = NC) (Figure S8) among women never diagnosed with HL in two cohort studies^42^, ^53^ of 7 941 388 births. The RRs, however, adjusted for maternal age and parity, for both PROM (1.17, 95% CI 0.73–1.89, *p* = 0.51) and PPH (1.03, 95% CI 0.62–1.69, *p* = 0.92) were not statistically significant.^42^

The pooled proportion of the two studies of women diagnosed with HL during pregnancy that included the outcome of anaemia was 69% (46 events, 67 births, 95% CI 57–80%, *I* ^2^ = NC, *p* = NC),^54,66^ compared with 4% (1099 events, 25 000 births, 95% CI 4–5%, *I* ^2^ = NC, *p* = NC) in one study that included pregnant women never diagnosed with HL (Figure S9).^42^ Neither study stated the proportion of women with anaemia according to the timing of therapy.

Pooled proportions in the group treated for HL before pregnancy and the group diagnosed with HL during pregnancy were not increased compared with the group never diagnosed with HL for outcomes of 5‐min Apgar score of <7, LBW, SGA, neonatal death, PIH, pre‐eclampsia, gestational diabetes, elective termination, stillbirth, CS and IOL (Figures S10–S20). There were also no statistically significant differences in aOR for these outcomes (Table 1). Regarding the timing of therapy, there were no significant differences in the proportions of CS, IOL, LBW and SGA (Figures S21–S24) among pregnant women who received antenatal therapy, compared with women who did not.

### 3.4 Subgroup analysis

The results of subgroup analysis for each outcome according to study design, region and risk of bias are summarised in Tables S3–S19 (Appendix S6). In the group diagnosed with HL during pregnancy, the proportion of PTB was 50% for studies in Europe (two studies, 12 events, 24 births, 95% CI 29–71%, *I* ^2^ = NC, *p* = NC),^61^, ^71^ compared with 13% for studies in the Americas (nine studies, 132 events, 901 births, 95% CI 6–20%, *I* ^2^ = 74.24%, *p* = 0.00) (Table S3).^48^, ^53^, ^54^, ^63^, ^65^, ^66^, ^70^, ^72^, ^73^ PTB rates did not significantly differ according to study design or risk of bias. There were no significant differences in subgroup analysis in the proportions of congenital malformations (Table S4) or miscarriage (Table S5).

In secondary outcome analysis, in the group treated for HL before pregnancy, the pooled proportion of women who had a CS was higher in cohort studies with a low risk of bias (three studies, 193 events, 625 births, 29%, 95% CI 20–39%, *I* ^2^ = NC, *p* = NC),^42^, ^47^, ^56^ compared with case series with a low–moderate risk of bias (two studies, four events, 55 births, 4%, 95% CI 0–12%, *I* ^2^ = NC, *p* = NC) (Table S6).^46^, ^49^ There were no significant differences in subgroup analysis of the remaining secondary outcomes (Tables S7–S19).

### 3.5 Sensitivity analysis

The results of sensitivity analyses are described in Appendix S7. There were no significant differences in these analyses compared with the original meta‐analyses of proportions (Tables S20 and S21).

### 3.6 Narrative synthesis results

A cohort study of 638 women diagnosed with HL during pregnancy in Canada conducted by El‐Messidi et al. (*n* = 7 916 388) reported a statistically significantly increased aOR for risk of blood transfusion (55 events, aOR 1.38, 95% CI 1.05–1.82, *p* = 0.023) and VTE (four events, aOR 7.93, 95% CI 2.97–21.22, *p* < 0.001), and a non‐statistically significant aOR for chorioamnionitis (ten events, aOR 0.91, 95% CI 0.48–1.69, *I* ^2^ = NC) (Table 1).^53^

### 3.7 Bias and heterogeneity

Heterogeneity among groups, ranging from substantial to considerable, was present in the meta‐analyses for all outcomes. In subgroup analysis, although heterogeneity was reduced, at least moderate heterogeneity remained for most outcomes, whereas for others, there were insufficient studies in each subgroup category to calculate the *I* ^2^ statistic. The majority of studies (30/33) were classified as having a low or low–moderate risk of bias (Tables S22–S23), although the GRADE certainty of findings for all outcomes were very low or low (Appendix S9).

## 4 DISCUSSION

### 4.1 Main findings

This systematic review examined maternal and perinatal outcomes among pregnant women treated for HL before pregnancy or with a diagnosis of HL during pregnancy. Rates of congenital malformations and miscarriage were not significantly increased among pregnant women with HL or a history of HL compared to women never diagnosed with HL. Proportions of PTB and anaemia were increased in the group diagnosed with HL during pregnancy, as were the aORs for blood transfusion and VTE. The rates of all other outcomes, including meta‐analyses according to timing of therapy, were not significantly increased compared with the general pregnant population.

### 4.2 Strengths and limitations

This systematic review had several strengths. It was based on a preprepared protocol and MOOSE guidelines were followed throughout. The inclusion of case series in this systematic review expands the pool of data compared with previous reviews of pregnancy outcomes that included HL as an exposure. Performing a systematic search of five databases supplemented by the manual review of reference lists ensured the comprehensive identification of eligible studies. Study selection, data extraction and risk‐of‐bias assessment performed independently by two reviewers reduced individual bias. By contacting authors directly for more information and including case series, abstracts and articles not published in English, efforts were made to ensure that this systematic review was as comprehensive as possible. The inclusion of subgroup and sensitivity analyses that demonstrated results comparable with the main meta‐analyses also increased our confidence in the results.

The study had some limitations. Only published original studies were included in this systematic review, and the exclusion of grey literature may have resulted in the omission of relevant data. Despite contacting authors, we were unable to obtain data specific to HL for some studies. The lack of a comparison group in case series and the lack of peer review for letters to the editor may have introduced a higher risk of bias and confounding.

Only observational studies that met the inclusion criteria for this review were identified. Given the nature of the review question, no randomised controlled trials exist on this topic, resulting in lower levels of evidence and more heterogeneous studies. Although most studies had a low or low–moderate risk of bias, the GRADE certainty for the findings were low or very low, and the heterogeneity levels were high, limiting the ability to draw concrete conclusions from the study.

Only a small number of studies, with low numbers of pregnancies/births, were available for most of the outcomes, thereby limiting the possibility of making robust conclusions, which is reflected in several estimates that are not statistically significant, but with associations that could not be ruled out. Although a separate analysis was performed according to the timing of therapy, several studies did not report these data. The studies included also did not present outcome data separately according to HL stage or treatment received, which may have influenced the outcomes. Although most studies considered several confounding factors, these differed between studies, resulting in heterogeneity in adjusted estimates. Some studies included wide study periods (for example the British Childhood Cancer Study included the years 1940–1991),^42^ and maternal care is likely to have improved throughout these time periods, which may have influenced the study outcomes. Details of maternal and perinatal care and socio‐economic status are also absent from many of the included studies, which also limits the interpretation of the results. The lack of distinction between elective and emergency CS and spontaneous and planned PTB is another limitation, as this prevents any differentiation between the direct effect of HL on pregnancy outcomes and the influence of an HL diagnosis on obstetric care.

### 4.3 Interpretation

The proportions of congenital malformations among women treated for HL before pregnancy and among women diagnosed with HL during pregnancy in our study were comparable with the general population of pregnant women.^74^, ^75^, ^76^ Miscarriage rates in the group diagnosed with HL during pregnancy were lower than the reported rates of 8%–15% among clinically recognised pregnancies.^77^, ^78^ This remained the case when a sensitivity analysis was performed that excluded studies not specifying the gestation or trimester at diagnosis.^30^ Several studies, however, only reported trimester at diagnosis rather than gestational age,^44^, ^64^, ^66^, ^71^ and therefore some pregnant women diagnosed in the late second trimester (25–27 weeks of gestation) included in the analysis may no longer have been at risk of miscarriage, resulting in the proportion of miscarriage being underestimated. Only one study reported aOR for miscarriage among women diagnosed with HL during pregnancy, which was not statistically significant (0.38, 95% CI 0.05–2.72).^53^

The higher proportion of PTB in patients diagnosed with HL during pregnancy compared with women never diagnosed with HL may have been linked to planned, rather than spontaneous, PTB. Planned PTB may have been increased in this group because clinicians and/or pregnant women may have wished to avoid treatment for HL during pregnancy. Increased PTB rates have also been reported in systematic reviews of pregnant women with acute promyelocytic leukaemia and acute myeloid leukaemia.^23^ ^,^ ^24^ The higher proportion of PTB in the group diagnosed with HL during pregnancy in Europe than in the Americas may reflect differing medical practices. The higher proportion of CS in cohort studies than in case series suggests that the true proportion of CS in the group with a history of HL may have been higher than that calculated in the overall meta‐analysis, as cohort studies are a more robust study design given their inclusion of a comparison group.^79^ Interpretation is limited, however, given the lack of distinction between elective and emergency CS.

We found no increased rates of adverse outcomes among pregnant women previously treated for HL. The increased aOR of VTE among women diagnosed with HL during pregnancy compared with the general pregnant population is likely to be related to the prothrombotic effects of malignancy and chemotherapy, which can activate the coagulation cascade.^80^ The higher pooled proportion of anaemia in the group diagnosed with HL during pregnancy compared with the group never diagnosed with HL is likely to be caused by cancer‐mediated overexpression of pro‐inflammatory cytokines, increased reactive oxygen species, the inhibition of erythropoiesis and chemotherapy‐induced myelosuppression.^81^

As a result of limitations in the currently published literature, including the small numbers of studies containing low numbers of affected pregnancies and the limited study quality, it is not possible to make robust conclusions, and associations cannot be confidently determined for some outcome measures, demonstrating the need for further research in this area.

## 5 CONCLUSION

We found no increased rates of most adverse pregnancy outcomes, including congenital malformations and miscarriage, among women with a current or previous diagnosis of HL compared with the general pregnant population. There were no statistically significant differences according to the timing of therapy. Although the group diagnosed with HL during pregnancy in our study had a higher PTB rate, which may have been linked to planned deliveries and an increased likelihood of VTE, anaemia and blood transfusion, our interpretation of the findings is limited because of the small numbers of studies and the low to very low GRADE certainty of the findings, which preclude firm conclusions.

Future primary research should be based on large cohorts of pregnant women with a current or previous diagnosis of HL and should stratify the outcome data by timing and treatment received, parity, race, and ethnicity. Data should also be reported separately for elective and emergency CS and planned and spontaneous PTB to further inform management decisions during pregnancy.

## AUTHOR CONTRIBUTIONS

OH and AK conceived the study and designed the protocol. OH performed the literature search. OH and DB selected the studies, extracted the relevant information and appraised the quality of the included studies. OH synthesised the data and wrote the first draft of the article. OH, AK, DB, GM and FMcC critically revised successive drafts of the article. All authors approve the final version of the article and accept responsibility for the article as published.

## FUNDING INFORMATION

OH received funding from the Specialist Training Fund for Higher Specialist Trainees, the Non‐Consultant Hospital Doctor (NCHD) Training Supports Scheme and St Luke's Radiation Oncology Network, Dublin.

## CONFLICT OF INTERESTS

None declared. Completed disclosure of interests form available to view online as supporting information.

## ETHICS APPROVAL

Ethical approval was granted by the Social Research Ethics Committee of the School of Public Health at University College Cork on 8 February 2022.

## Supporting information


Appendix S1.

Appendix S2.

Appendix S3.

Appendix S4.

Appendix S5.

Appendix S6.

## Data Availability

Data available in article supplementary material.
