# Supplementary material for: Maternal and perinatal outcomes following a diagnosis of Hodgkin lymphoma during or prior to pregnancy: A systematic review
Source: BJOG. 2022 Dec 12;130(4):336–47. doi: 10.1111/1471-0528.17347 (PMC10107208; doi:10.1111/1471-0528.17347)
Supplement: Supplementary file 1 — Appendix S1. [file BJO-130-336-s005.docx]

**Appendices**

**Appendix A:** Protocol for systematic review.

**Appendix B:** Search terms for each electronic database.

**Appendix C:** Flow diagram of search strategy and selection of studies for inclusion in systematic review.

**Appendix D:** Summary of characteristics of included studies:

- **Table S1.** Summary of characteristics of studies of patients diagnosed with and treated for Hodgkin lymphoma (HL) before pregnancy.
- **Table S2.** Summary of characteristics of studies of patients with Hodgkin lymphoma during pregnancy.

**Appendix E:** Proportion meta-analysis forest plots for included studies

- **Figure S1.** Proportion meta-analysis forest plot (random effects) for congenital malformations.
- **Figure S2.** Proportion meta-analysis forest plot (random effects) for congenital malformations by timing of therapy.
- **Figure S3.** Proportion meta-analysis forest plot (random effects) for preterm birth.
- **Figure S4.** Proportion meta-analysis forest plot (random effects) for preterm birth by timing of therapy.
- **Figure S5.** Proportion meta-analysis forest plot (random effects) for miscarriage.
- **Figure S6.** Proportion meta-analysis forest plot (random effects) for miscarriage by timing of therapy.
- **Figure S7.** Proportion meta-analysis forest plot (random effects) for premature rupture of membranes.
- **Figure S8.** Proportion meta-analysis forest plot (random effects) for postpartum haemorrhage.
- **Figure S9.** Proportion meta-analysis forest plot (random effects) for anaemia.
- **Figure S10.** Proportion meta-analysis forest plot (random effects) for 5-minute Apgar score < 7.
- **Figure S11.** Proportion meta-analysis forest plot (random effects) for low birth weight.
- **Figure S12.** Proportion meta-analysis forest plot (random effects) for small for gestational age.
- **Figure S13.** Proportion meta-analysis forest plot (random effects) for neonatal death.
- **Figure S14.** Proportion meta-analysis forest plot (random effects) for pregnancy-induced hypertension.
- **Figure S15.** Proportion meta-analysis forest plot (random effects) for pre-eclampsia.
- **Figure S16.** Proportion meta-analysis forest plot (random effects) for gestational diabetes.
- **Figure S17.** Proportion meta-analysis forest plot (random effects) for elective termination of pregnancy.
- **Figure S18.** Proportion meta-analysis forest plot (random effects) for stillbirth.
- **Figure S19.** Proportion meta-analysis forest plot (random effects) for caesarean section.
- **Figure S20.** Proportion meta-analysis forest plot (random effects) for induction of labour.
- **Figure S21.** Proportion meta-analysis forest plot (random effects) for low birth weight by timing of therapy.
- **Figure S22.** Proportion meta-analysis forest plot (random effects) for small for gestational age by timing of therapy.
- **Figure S23.** Proportion meta-analysis forest plot (random effects) for caesarean section by timing of therapy.
- **Figure S24.** Proportion meta-analysis forest plot (random effects) for induction of labour by timing of therapy.

**Appendix F:** Subgroup analyses:

- **Table S3.** Subgroup meta-analysis of proportions for preterm birth.
- **Table S4.** Subgroup meta-analysis of proportions for congenital malformations.
- **Table S5.** Subgroup meta-analysis of proportions for miscarriage.
- **Table S6.** Subgroup meta-analysis of proportions for caesarean section.
- **Table S7.** Subgroup meta-analysis of proportions for premature rupture of membranes.
- **Table S8.** Subgroup meta-analysis of proportions for postpartum haemorrhage.
- **Table S9.** Subgroup meta-analysis of proportions for anaemia.
- **Table S10.** Subgroup meta-analysis of proportions for 5-minute APGAR score < 7.
- **Table S11.** Subgroup meta-analysis of proportions for low birth weight.
- **Table S12.** Subgroup meta-analysis of proportions for small for gestational age.
- **Table S13.** Subgroup meta-analysis of proportions for neonatal death.
- **Table S14.** Subgroup meta-analysis of proportions for pregnancy-induced hypertension.
- **Table S15.** Subgroup meta-analysis of proportions for pre-eclampsia.
- **Table S16.** Subgroup meta-analysis of proportions for gestational diabetes.
- **Table S17.** Subgroup meta-analysis of proportions for elective termination of pregnancy.
- **Table S18.** Subgroup meta-analysis of proportions for stillbirth.
- **Table S19.** Subgroup meta-analysis of proportions for induction of labour.

**Appendix G: Sensitivity meta-analyses**

- **Table S20.** Sensitivity meta-analysis with studies containing heterogeneous outcome data excluded.
- **Table S21.** Sensitivity meta-analysis with studies published prior to 2000 and 1990 excluded.

**Appendix H:** Risk of bias assessment:

- **Table S22.** Level of bias in studies of patients diagnosed with and treated for Hodgkin lymphoma before pregnancy.
- **Table S23.** Level of bias in studies of patients with Hodgkin lymphoma during pregnancy.

**Appendix I:** Certainty of evidence assessed using the GRADE approach for each outcome.

**Appendix A:** Protocol for systematic review

**Review question**

Is a previous or current diagnosis of Hodgkin lymphoma (HL) associated with adverse maternal and perinatal outcomes?

**Aim**

The aim of this systematic review is to provide a comprehensive summary of the published literature regarding maternal and perinatal outcomes following a diagnosis of Hodgkin lymphoma prior to or during pregnancy.

Objectives

1. To assess if Hodgkin lymphoma diagnosed and treated before pregnancy is associated with increased adverse maternal and perinatal outcomes compared to the general population.
2. To assess if Hodgkin lymphoma diagnosed and treated during pregnancy is associated with increased adverse maternal and perinatal outcomes compared to the general population.
3. To determine if treatment for Hodgkin lymphoma diagnosed during pregnancy should be deferred until after delivery.

**Methods**

The MOOSE (Meta-analyses Of Observational Studies in Epidemiology) reporting tool will be used when conducting this systematic review.

**Information sources**

OH will independently conduct a systematic literature search of the following electronic databases from inception: PubMed/Medline, Cochrane library, Scopus, Embase and Science Direct. Reference lists of all retrieved articles studies will also be manually searched to identify additional studies for inclusion.

**Search strategy**

Search terms will include relevant medical subject headings (MeSH) and keywords. Appendix C describes the search terms to be used. Search terms #3 to #33 will be combined using ‘OR’ to form search term #33 (outcomes). The initial search will consist of #1 (population) AND #2 (exposure) AND #33 (outcomes). These search terms will be formatted for use in each named electronic database.

All original studies except case studies pertaining to maternal and/or perinatal outcomes following a diagnosis of Hodgkin lymphoma during or prior to pregnancy will be included. There will be no restriction on language, provided an English translation is available, location of study or publication date. Where full text is not available by search, corresponding authors will be contacted directly by email. Searches will be repeated prior to final analysis and any additional relevant studies identified will be retrieved for inclusion.

EndNote^TM^ 20 reference management software will be used to store search results and remove duplicates. Study selection will be independently performed by OH and DB. AK will be involved in the discussion of any papers where consensus on eligibility cannot be achieved.

**Participants/population**

Pregnant women and their children

**Intervention(s), exposure(s)**

Previous or current diagnosis and/or treatment of Hodgkin lymphoma

**Comparator(s)/control**

No previous or current diagnosis of Hodgkin lymphoma

**Types of study to be included**

Original studies including randomised controlled trials, cohort studies, case-control studies, cross-sectional studies and case series.

**Main outcome(s)**

*Pregnancy outcomes:* Miscarriage, elective termination of pregnancy, stillbirth

*Labour and delivery characteristics:* Premature rupture of membranes, induction of labour, caesarean section

*Maternal outcomes:* Pregnancy-induced hypertension, pre-eclampsia, gestational diabetes, anaemia, postpartum haemorrhage, blood transfusion, chorioamnionitis, venous thromboembolism

*Perinatal outcomes:* Preterm birth, low birth weight, small for gestational age, congenital malformations, 5-minute Apgar score < 7, neonatal death

**Data extraction (selection and coding)**

Data collected will include study title, author(s), country, journal, year of publication, study period, type of study, sample size, timing of Hodgkin lymphoma diagnosis, treatment characteristics and outcomes. Corresponding authors will be contacted directly by email for any missing data or if additional data to that published are required. Data will be recorded in an excel spreadsheet.

**Risk of bias (quality) assessment**

The quality of included studies will be assessed using the an appropriate quality assessment tool described by McDonald et al. The Grading of Recommendations, Assessment, Development and Evaluations (GRADE) framework will be used to evaluate certainty of results of meta-analysis. If appropriate, sensitivity analyses will be performed to examine the effect of excluding studies with heterogeneous outcome data.

**Strategy for data synthesis**

A study flow diagram will be presented summarising the literature search and selection process of included studies. Explanations will be provided for excluded studies. Results from included studies will be presented in the form of summary tables and forest plots.

Where sufficient data are present, a meta-analysis of proportions will be conducted using Stata version 17 to pool results from included studies and calculate an overall estimate of the relationship between Hodgkin lymphoma and adverse maternal and perinatal outcomes*.* Pooled estimates of odds ratios with 95% confidence intervals will be calculated using Review Manager 5.4 for those studies for which odds ratios are provided or can be calculated. Heterogeneity between studies will be assessed using the I^2^ statistic. As there will likely be heterogeneity present between studies due to differences in study design, measurement and outcomes, random-effects models will be used to generate a summary estimate of the magnitude of effect. If data are insufficient for meta-analysis, a narrative synthesis will be performed.

**Analysis of subgroups or subsets**

Where appropriate, subgroup analyses will be performed according to study design, WHO region and risk of bias assessment.

**Appendix B.** Search strategy for each electronic database

| **Search terms (inception – June 15, 2022)** |
| --- |
| 1. Pregnan* |
| 1. Hodgkin* lymphoma |
| 1. Maternal complication* |
| 1. Perinatal complication* |
| 1. Prenatal complication* |
| 1. Obstetric complication* |
| 1. Pregnancy complication* |
| 1. Labo?r complication* |
| 1. Miscarriage |
| 1. Elective termination |
| 1. Abortion |
| 1. Stillbirth |
| 1. Live birth |
| 1. Premature rupture of membranes |
| 1. Induction of labo?r |
| 1. F?etal malpresentation |
| 1. C?esarean delivery |
| 1. Complications of labo?r |
| 1. Pregnancy-induced hypertension |
| 1. Pre-eclampsia |
| 1. Gestational diabetes |
| 1. An?emia |
| 1. Postpartum h?emorrhage |
| 1. Blood transfusion |
| 1. Chorioamnionitis |
| 1. Venous thromboembolism |
| 1. Preterm birth |
| 1. Low birth weight |
| 1. Small for gestational age |
| 1. Intrauterine growth restriction |
| 1. Congenital malformation |
| 1. F?etal distress |
| 1. [#3 or #4 or #5 or #6 or #7 or #8 or #9 or #10 or #11 or #12 or #13 or #14 or #15 or #16 or #17 or #18 or #19 or #20 or #21 or #22 or #23 or #24 or #25 or #26 or #27 or #28 or #29 or #30 or #31 or #32] |
| 1. [#1 and #2 and #33] |

**Appendix C.** Flow diagram of search strategy and selection of studies for inclusion in systematic review.

Papers identified through database search n = 6,139

Records after duplicates removed n = 5,527

Records removed after reviewing titles and abstracts n = 5,446

Full text articles reviewed n = 76

1 eligible vignette identified

1 eligible letter to the editor

Authors of 3 conference abstracts contacted for data specific to HL – 1 response received but no additional data provided

Records removed after reviewing full texts n = 50

6 Review article, not original study

16 Did not include outcomes of interest

1 did not include exposure of interest

1 data not sufficient to compute estimates

14 Ineligible study design (case report)

8 Older versions of included publications

Authors of 5 papers contacted for data specific to HL – 1 response with requested data

Eligible full text articles found n = 26

Additional articles identified following hand-search of reference lists n = 5

Total number of records included in systematic review n = 33 (31 full papers, 1 vignette, 1 letter to the editor)

**Appendix D.** Summary of characteristics of included studies.

**Table S1.** Summary of characteristics of studies of patients diagnosed with and treated for Hodgkin lymphoma before pregnancy.

| **Study** | **Data source** | **Study design** | **Region, study period** | **Number of female HL survivors** | **Number of pregnancies** | **Outcome measures** | **Assessment method of outcome** | **Main findings** |
| --- | --- | --- | --- | --- | --- | --- | --- | --- |
| Anderson et al., 2017 ^47^ | North Carolina Central Cancer Registry | Cohort study | United States, 2000-2013 | 179 | Not reported | CS PTB LBW  SGA  APGAR < 7 | Central Cancer Registry records linked to statewide birth certificate files | aRR (95% CI): CS: 1.08 (0.88-1.34) PTB: 1.59 (1.06-2.37) LBW: 1.44 (0.89-2.33) SGA: 1.08 (0.71-1.64) APGAR < 7: 0.92 (0.30-2.79) |
| Hartnett et al., 2017 ^56^ | Cancer registries in Georgia, North Carolina and Tennessee, United States | Cohort study | United States, 1994-2012 | 293 | 293 | CS PTB  LBW  SGA  APGAR < 7 | Cancer registry and birth certificate data linked to vital records. | aRR (95% CI): CS: 1.1 (1.0-1.3) PTB: 1.1 (0.7-1.5) LBW: 1.0 (0.6-1.5) SGA: 1.0 (0.7-1.4) APGAR < 7: 3/293 = 1.0% |
| Reulen et al., 2017 ^42^ | British Childhood Cancer Survivor Study | Cohort study | United Kingdom, 1940-1991 | 93 | 153 | CS PIH  Gestational DM  Anaemia PROM  Foetal distress PPH | National Registry of Childhood Tumours and Hospital Episode Statistics for England. | aRR (95% CI):  Elective CS: 0.59 (0.32-1.08);  Emergency CS: 0.59 (0.36-0.97) PIH: 0.61 (0.23-1.58) Gestational DM: 1.33 (0.46-3.90) Anaemia: 1.12 (0.59-2.13) PROM: 1.17 (0.73 -1.89) Foetal distress: 0.73 (0.52-1.02) PPH: 1.03 (0.62-1.69) |
| De Sanctis et al., 2012 ^51^ | Department of Hematology and Radiotherapy, University “Sapienza” of Rome, Italy | Case series | Italy, 1972-1999 | 99 | 145 | Miscarriage Stillbirth PTB  LBW Congenital malformations | Medical records | aOR (95% CI) according to type of treatment received:  Miscarriage: CRT: 9/90 0.11 (0.055-0.2); RT alone: 3/42 0.077 (0.024-0.249); CT alone: 1/13 0.083 (0.010-0.641), p = 0.85.  PTB: CRT: 6/90 0.071 (0.031-0.164); RT alone: 2/42 0.005 (0.012-0.207); CT alone: 1/13 0.083 (0.011-0.641), p = 0.89.  LBW: CRT: 3/90 0.047 (0.015-0.149), p = 0.40.  Stillbirth: 0/132 = 0% Congenital malformations: CRT: 2/134 = 1.5% |
| Reulen et al., 2009 ^43^ | British Childhood Cancer Survivor Study | Cohort study | United Kingdom, 1940-1991 | Not reported | 346 | Miscarriage Elective termination Stillbirth PTB  LBW | National Registry of Childhood Tumours and patient questionnaires | aOR (95% CI):  Miscarriage: 0.8 (0.5-1.3) Elective termination: 1.1 (0.7-1.8) Stillbirth: 1.4 (0.3-5.7) PTB: 0.9 (0.5-1.6) LBW: 1.0 (0.4-2.3) |
| Langagergaard et al., 2008 ^61^ | Danish Cancer Registry and Birth Registry | Cohort study | Netherlands, 1973-2002 | 192 | 192 | Stillbirth PTB  LBW Congenital malformations | Danish Cancer Registry (ICD-7) and Birth Registry (ICD-8 and ICD-10) | aOR (95% CI): Stillbirth: 2.0 (0.3-15.4) PTB: 1.1 (0.6-2.0) LBW: 0.6 (0.2-2.6) Congenital malformations: 1.7 (0.9-3.1) |
| Signorello et al., 2006 ^12^ | Childhood Cancer Survivor Study | Cohort study | United States, 1968-2002 | Not reported | 337 | PTB  LBW  SGA | Medical records | PTB: Cases: 65/337 = 19.2% Controls: 145/1152 = 12.6% LBW: Cases: 20/337 = 5.9% Controls: 48/1142 = 4.2% SGA: Cases: 30/337 = 9.0% Controls: 101/1103 = 9.2% |
| Roman et al., 2005 ^67^ | United Kingdom Childhood Cancer Study | Cohort study | United Kingdom, 1992-1996 | 63 | 61 | Pre-eclampsia Anaemia | Patient medical records | aOR (95% CI): Pre-eclampsia: 0.7 (0.2-1.8) Anaemia: 1.0 (0.3-2.7) |
| Green et al., 2002 ^55^ | Childhood Cancer Survivor Study | Cohort study | United States, 1970-1986 | Not reported | 1082 | Miscarriages Elective termination Stillbirth | Medical records and patient questionnaires | cRR (95% CI): Miscarriage: 1.10 (0.87-1.40) Elective termination: 1.32 (1.02-1.71) Stillbirth: 1.60 (0.64-4.03) |
| Brierley et al., 1998 ^50^ | Princess Margaret Hospital, Toronto, Canada | Case series | Canada, 1973-1984 | 54 | 134 | Miscarriage Elective termination Congenital malformations | Self-reported questionnaire | Miscarriage: 20/134 = 14.9% Elective termination: 19/134 = 14.2% Congenital malformations: 1/95 = 1.1% |
| Swerdlow et al., 1996* ^69^ | Mount Vernon Hospital, Middlesex, England | Cohort study | United Kingdom, 1970-1991 | 26 | Not reported (pregnancies from male and female survivors reported together) | Miscarriage Elective termination Stillbirth PTB  LBW Congenital malformations | Medical records and patient questionnaires | cRR (95% CI): PTB: 0.88 (0.32-2.46) LBW: 1.58 (0.52-4.26)  Miscarriage: 6/26 = 23.1% Elective termination: 4/26 = 15.4% Stillbirth: 0/26 = 0% Congenital malformations: 3/26 = 11.5% |
| Aisner et al., 1993 ^46^ | University of Maryland Cancer Centre, Maryland, United States | Case series | United States, 1965-1985 | 35 | 54 | Miscarriage Elective termination  Caesarean delivery Stillbirth PTB Congenital malformations | Self-reported questionnaire and interviews | Miscarriage: 1/54 = 1.9% Elective termination: 9/54 = 16.7% Caesarean delivery: 3/44 = 6.8% Stillbirth: 2/44 = 4.5% PTB: 0/42 = 0% Congenital malformations: 6/54 = 11.1% |
| Janov et al., 1992 ^59^ | Cancer and Leukemia Group B | Cohort study | United States, 1966-1986 | 16 | 16 | Neonatal death LBW | Telephone questionnaire | cRR (95% CI):  Neonatal death: 2.5 (0.3-9.0)  LBW: 0/15 |
| [Balcewicz-Sablińska et al. , 1990](https://pubmed.ncbi.nlm.nih.gov/?size=200&term=Balcewicz-Sabli%C5%84ska+K&cauthor_id=2260411) ^49^ | Institute of Haematology and Blood Transfusion, Warsaw, Poland | Case series | Poland, not reported | 8 | 11 | Miscarriage Elective termination CS Stillbirth Neonatal death LBW Congenital malformations | Medical records and patient questionnaires | Miscarriage: 0/11 = 0% Elective termination: 0/11 = 0% CS: 1/11 = 9.1% Stillbirth: 0/11 = 0% Neonatal death: 0/11 = 0% LBW: 0/11 = 0% Congenital malformations: 0/11 = 0% |
| Lacher et al., 1986 ^60^ | Memorial Sloan-Kettering Cancer Centre, New York, United States | Case series | United States, 1973-1981 | 12 | 17 | Miscarriage Elective termination Stillbirth Neonatal death | Patient questionnaires, medical records, direct personal patient communication | Miscarriage: 0/16 = 0% Elective termination: 4/16 = 25% Stillbirth: 0/12 = 0% Neonatal death: 0/12 = 0% |
| Slanina et al., 1985 ^68^ | Freiburg im Breisgau | Case series | Germany, 1949-1981 | 122 | 63 | Miscarriage Elective termination PTB  Congenital malformations | Medical records and patient questionnaires | Miscarriage: 5/63 = 7.9% Elective termination: 12/63 = 19.0% PTB: 3/46 = 6.5% Congenital malformations: 2/46 = 4.3% |
| McKeen et al., 1979 ^72^ | Cancer and Leukemia Group B | Case series | United States, not reported | 44 | 44 | Miscarriage  Elective termination Stillbirth PTB/LBW Congenital malformations | Communications with patients and physicians | Miscarriage:4/44 = 9.1% Elective termination: 4/44 = 9.1% Stillbirth: 2/36 = 5.6% Congenital malformations: 5/36 = 13.9% 6 infants were delivered prematurely or had LBW |
| Holmes et al., 1978 ^57^ | University of Kansas Medical Centre, Kansas, United States | Cohort study | United States, 1944-1975 | 48 | 93 | Miscarriage Elective termination Stillbirth PTB  Neonatal death Congenital malformations | Medical records | Miscarriage: Cases: 7/93 = 7.5%; Controls: 20/228 = 8.8% Elective termination: Cases: 3/93 = 3.2%; Controls: 0/93 = 0% Stillbirth: Cases: 2/82 = 2.4%; Controls: 3/207 = 1.4% PTB: Cases: 2/82 = 2.4%; Controls: 3/207 = 1.4% Neonatal death: Cases: 0/82 = 0%; Controls: 2/206 = 1.0% Congenital malformations: Cases: 8/84 = 9.5%; Controls: 26/209 = 12.4% |
| Le Floch et al., 1976 ^62^ | Stanford University Medical Centre, California, United States | Case series | United States, 1968-1976 | 9 | 10 | Miscarriage Elective termination Congenital malformations | Medical records | Miscarriage: 1/10 = 10% Elective termination: 2/10 = 20% Congenital malformations: 0/8 = 0% |

*results include figures for both female survivors of HL and female partners of male survivors of HL.

Abbreviations: APGAR < 7, 5-minute APGAR score < 7; CS, caesarean section; CT, chemotherapy; CRT, chemoradiotherapy; DM, diabetes mellitus; HL, Hodgkin lymphoma, LBW, low birth weight; aOR: adjusted odds ratio; cOR: crude odds ratio; PIH, pregnancy-induced hypertension; PPH, postpartum haemorrhage; PTB, preterm birth; PROM, premature rupture of membranes; aRR: adjusted relative risk; cRR crude relative risk; SGA, small for gestational age.

**Table S2.** Summary of characteristics of studies of patients with Hodgkin lymphoma during pregnancy.

| **Study** | **Data source** | **Study design** | **Region, study period** | **Number of women with HL in pregnancy** | **Number of pregnancies** | **Outcomes studied** | **Assessment Method of Outcome** | **Main findings** |
| --- | --- | --- | --- | --- | --- | --- | --- | --- |
| Kroll-Balcerzak et al. 2019 ^71^ | Department of Hematology and Bone Marrow Transplantation Poznan University of Medical Sciences, Poland | Case series | Poland, 1997-2014 | 9 | 9 | Miscarriage Elective termination PTB  LBW  APGAR < 7 | Not reported | Miscarriage: 0/9 = 0% Elective termination: 0/9 = 0% PTB: 4/9 = 44.4% LBW: 1/9 = 11.1% APGAR < 7 : 0/9 = 0% |
| Maggen et al., 2019 ^64^ | International Network on Cancer, Infertility and Pregnancy (INCIP) database | Cohort study | Multinational: United States, Russia, Belgium, Netherlands, Mexico, Italy, Czech Republic, Israel, United Kingdom, Greece, Denmark, 1969-2018 | 134 | 134 | Miscarriage Elective termination Stillbirth PTB  LBW  SGA  Congenital malformations | INCIP database | Miscarriage: 2/108 = 1.9% Elective termination: 10/134 = 7.5% Stillbirth: 2/134 = 1.5% PTB: Antenatal CT: 24/69 = 34.8% No CT: 21/42 = 50% LBW: Antenatal CT: 21/69 = 30.4% No CT: 11/42 = 26.2% SGA: Antenatal CT: 15/69 = 21.7% No CT: 6/42 = 14.3% Congenital malformations: Antenatal CT: 2/69 = 2.9% No CT: 1/42 = 2.4% |
| Niu et al., 2019 ^38, 73^ | Washington State-linked vital-hospital discharge records, Washington, United States | Cohort study | United States, 1987-2012 | 95 | 95 | CS  PTB  LBW  SGA  APGAR < 7 Pre-eclampsia/eclampsia Gestational DM  Foetal distress | Washington State-linked vital-hospital discharge records. | CS: Cases: 9/41 = 22.0%; Controls: 14626/57203 = 25.6% PTB: Cases: 13/92 = 14.1%; Controls: 9478/138096 = 6.9% LBW: Cases: 9/94 = 9.6%; Controls: 6276/138735 = 4.5% SGA: Cases: 11/88 = 12.5%; Controls: 11501/127520 = 9.0% APGAR < 7: Cases: 8/93 = 8.6%; Controls: 5695/138626 = 4.1%  Pre-eclampsia/eclampsia: Cases: 7/94 = 7.4%; Controls: 8336/139098 = 6.0% Gestational DM: Cases: 5/94 = 5.3%; Controls: 8261/139098 =5.9% Foetal distress: Cases: 10/94 = 10.6%; Controls: 14954/139098 = 10.8% |
| Avilés et al., 2018 ^48^ | Oncology Hospital National Medical Center, Mexico | Case series | Mexico, 1988-2013 | 44 | 44 | Miscarriage Elective termination PTB Neonatal death LBW Congenital malformations | Medical records | Miscarriage: 0/44 = 0% Elective termination: 0/44 = 0% PTB: 10/44 = 22.7% Neonatal death: 0/44 = 0% LBW: 4/44 = 9.1% Congenital malformations: 0/44 = 0% |
| Pinnix et al., 2016 ^66^ | MD Anderson Cancer Center, Texas, United States | Case series | United States, 1991-2014 | 31 | 31 | Miscarriage Elective termination PTB  Anaemia PROM | Medical records | Miscarriage: 3/31 = 9.7% Elective termination: 2/31 = 6.5% PTB: 8/26 = 30.8% Anaemia: 20/27 = 74% PROM: 0/26 = 0% |
| El-Messidi et al., 2015 ^53^ | Healthcare Cost and Utilization Project-Nationwide Inpatient Sample | Cohort study | Canada, 2003-2011 | 638 | 638 | Miscarriage IOL  CS  PTB  IUGR  Congenital malformations PIH  Pre-eclampsia Gestational DM  PROM  PPH  Blood transfusion Chorioamnionitis VTE | Healthcare Cost and Utilization Project-Nationwide Inpatient Sample | Miscarriage: cOR 0.38 (0.05-2.67) aOR 0.38 (0.05-2.72) IOL: cOR 1.28 (0.69-2.40) aOR 1.28 (0.68-2.38) CS: cOR 1.18 (1.0-1.39) aOR 1.14 (0.97-1.34)  PTB: cOR 1.99 (1.58-2.50) aOR 1.93 (1.53-2.42) IUGR: cOR 1.20 (0.72-1.99) aOR 1.17 (0.70-1.95) Congenital malformations: cOR 1.98 (0.82-4.77) aOR 1.72 (0.71-4.15) PIH: cOR 0.70 (0.41-1.18) aOR 0.66 (0.39-1.12) Pre-eclampsia: cOR 1.35 (0.95-1.91) aOR 1.30 (0.92-1.85) Gestational DM: cOR 1.29 (0.94-1.75) aOR 1.28 (0.94-1.75) PROM: 0 PPH: cOR 1.24 (0.76-2.0) aOR 1.25 (0.77-2.02) Blood transfusion: cOR 1.49 (1.13-1.96) aOR 1.38 (1.05-1.82) Chorioamnionitis: cOR 0.90 (0.48-1.68) aOR 0.91 (0.48-1.69) VTE: cOR 8.74 (3.27-23.36) aOR 7.93 (2.97-21.22) |
| Van Calsteren et al., 2015 ^45^ | Journal of Clinical Oncology | Case series | Multinational: Belgium, Netherlands, Czech Republic, 1998-2008 | 11 | 11 | SGA | Medical records | SGA: 2/11 = 18.2% |
| Evens et al., 2013 ^54^ | 11 academic centres in United States | Case series | United States, 1999-2011 | 40 | 40 | Miscarriage Elective termination IOL CS  PTB SGA  Congenital malformations Pre-eclampsia Gestational DM Anaemia PROM  PPH | Medical records | Miscarriage: 0/31 = 0% Elective termination: 3/40 = 7.5% IOL: Antenatal therapy: 7/20 = 35% No therapy: 5/11 = 45.5%  CS: Antenatal therapy: 4/20 = 20% No therapy: 2/11 = 18.2% PTB: Antenatal therapy: 9/24 = 37.5% No therapy: 5/13 = 38.5% SGA: Antenatal therapy: 2/10 = 10% No therapy: 2/7 = 28.6% Congenital malformations: 0/31 = 0% Pre-eclampsia: 0/31 = 0% Gestational DM: Antenatal therapy: 0/20 = 0% No therapy: 1/11 = 9.1% Anaemia: 26/40 = 65% PROM: Antenatal therapy: 0/20 = 0% No therapy: 1/11 = 9.1% PPH: Antenatal therapy: 2/20 = 10% No therapy: 0/11 = 0% |
| Langagergaard et al., 2008 ^61^ | Danish Cancer Registry and Birth Registry | Cohort study | Netherlands, 1973-2002 | 15 | 15 | Stillbirth PTB  LBW  Congenital malformations | Danish Cancer Registry (ICD-7) and Birth Registry (ICD-8 and ICD-10) | aOR (95% CI): Stillbirth: 0 PTB: 26.6 (8.5-83.0) LBW: 0 Congenital malformations: 2.7 (0.3-22.8) |
| Dilek et al. 2006 ^52^ | Yüzüncü Yıl University, Van, Turkey | Case series | Turkey, 1996-2003 | 5 | 6 | Miscarriage Elective termination  Stillbirth Neonatal death LBW IUGR Congenital malformations | Medical records | Miscarriage: 0/6 = 0% Elective termination: 1/6 = 16.7% Stillbirth: 1/5 = 20%  Neonatal death: 0/4 = 0% LBW: 2/4 = 50% IUGR: 2/4 = 50% Congenital malformations: 1/4 = 25% |
| Lishner et al., 1992 ^63^ | Princess Margaret Hospital, Toronto, Canada | Cohort study | Canada, 1958-1984 | 48 | 50 | Miscarriage Elective termination CS  Stillbirth PTB Neonatal death Congenital malformations | Medical records | Miscarriage: 5/23 = 21.7% Elective termination: 4/50 = 8% CS: Cases: 5/25 = 20% Controls: 6/38 = 15.8% Stillbirth: Cases: 2/40 = 5% Controls: 0/38 = 0% PTB: Cases: 1/29 = 3.4% Controls: 1/37 = 2.7% Neonatal death: Cases: 1/31 = 3.2% Controls: 0/38 = 0% Congenital malformations: Cases: 1/31 = 3.2% Controls: 0/38 = 0% |
| Woo et al., 1992 ^70^ | MD Anderson Cancer Center, Texas, United States | Case series | United States, 1956-1990 | 25 | 25 | Miscarriage Elective termination PTB  LBW  Congenital malformations | Medical records and patient questionnaires | Miscarriage: 0/16 = 0% Elective termination: 6/25 = 24% PTB: 0/16 = 0% LBW: 0/10 = 0% Congenital malformations: 0/16 = 0% |
| [Balcewicz-Sablińska et al., 1990](https://pubmed.ncbi.nlm.nih.gov/?size=200&term=Balcewicz-Sabli%C5%84ska+K&cauthor_id=2260411) ^49^ | Institute of Haematology and Blood Transfusion, Warsaw, Poland | Case series | Poland, not reported | 9 | 9 | Miscarriage Elective termination CS Stillbirth Neonatal death LBW Congenital malformations | Medical records and patient questionnaires | Miscarriage: 0/8 = 0% Elective termination: 0/9 = 0% CS: Antenatal therapy: 1/6 = 16.7% No therapy: 0/3 = 0% Stillbirth: 0/9 = 0% Neonatal death: 0/9 = 0% LBW: Antenatal therapy: 1/6 = 16.7% No therapy: 0/3 = 0% Congenital malformations: 0/9 = 0% |
| Nisce et al., 1986 ^65^ | Memorial Sloan-Kettering Cancer Centre, New York, United States | Case series | United States, 1969-1982 | 17 | 17 | Elective termination CS  Stillbirth PTB Congenital malformations | Medical records | Elective termination: 6/17 = 35.3% CS: 2/11 = 18.2% Stillbirth: 0/11 = 0% PTB: 0/11 = 0% Congenital malformations: 0/11 = 0%11 |
| Tawil et al., 1985 ^44^ | Hôpital Maisonneuve-Rosemont, Montreal, Canada | Case series | Canada, 1968-1979 | 12 | 12 | Miscarriage Elective termination IOL  CS Congenital malformations Pre-eclampsia | Medical records | Miscarriage: 0/5 = 0% Elective termination: 2/12 = 16.7% IOL: 1/10% CS: 1/10% Congenital malformations: 0/10% Pre-eclampsia: 1/10 = 10% |
| Jacobs et al., 1981 ^58^ | Stanford University Medical Centre, California, United States | Case series | United States, 1963-1979 | 15 | 15 | Miscarriage Elective termination IOL Congenital malformations | Medical records | Miscarriage: Antenatal therapy: 1/6 = 16.7% No therapy: 0/5 = 0% Elective termination: 6/15 = 40% IOL: Antenatal therapy: 0/6 = 0% No therapy: 1/2 = 50% Congenital malformations: 0/8 = 0% |
| McKeen et al., 1979 ^72^ | Cancer and Leukemia Group B | Case series | United States, not reported | 14 | 14 | Miscarriage Elective termination Stillbirth PTB/LBW Congenital malformations | Communications with patients and physicians | Miscarriage: 1/14 =7.1% Elective termination: 5/14 = 35.7% Stillbirth: 0/8 = 0% PTB/LBW: 0/8 = 0% Congenital malformations: 0/8 = 0% |

Abbreviations: APGAR < 7, 5-minute APGAR score < 7; CS, caesarean section; CT, chemotherapy; DM, diabetes mellitus; IOL, induction of labour; IUGR, intrauterine growth restriction; LBW, low birth weight; aOR: adjusted odds ratio; cOR: crude odds ratio; PIH, pregnancy-induced hypertension; PPH, postpartum haemorrhage; PTB, preterm birth; PROM, premature rupture of membranes; SGA, small for gestational age; VTE, venous thromboembolism.

**Appendix E.** Proportion meta-analysis forest plots for included studies.

**Figure S1.** Proportion meta-analysis forest plot (random effects) for congenital malformations.

**Figure S2.** Proportion meta-analysis forest plot (random effects) for congenital malformations by timing of therapy.

**Figure S3.** Proportion meta-analysis forest plot (random effects) for preterm birth.**Figure S4.** Proportion meta-analysis forest plot (random effects) for preterm birth by timing of therapy.

**Figure S5.** Proportion meta-analysis forest plot (random effects) for miscarriage.

**Figure S6.** Proportion meta-analysis forest plot (random effects) for miscarriage by timing of therapy.

**Figure S7.** Proportion meta-analysis forest plot (random effects) for premature rupture of membranes.

**Figure S8.** Proportion meta-analysis forest plot (random effects) for postpartum haemorrhage.

**Figure S9.** Proportion meta-analysis forest plot (random effects) for anaemia.

**Figure S10.** Proportion meta-analysis forest plot (random effects) for 5-minute Apgar score < 7.

**Figure S11.** Proportion meta-analysis forest plot (random effects) for low birth weight.

**Figure S12.** Proportion meta-analysis forest plot (random effects) for small for gestational age.

**Figure S13.** Proportion meta-analysis forest plot (random effects) for neonatal death.

**Figure S14.** Proportion meta-analysis forest plot (random effects) for pregnancy-induced hypertension.

**Figure S15.** Proportion meta-analysis forest plot (random effects) for pre-eclampsia.

**Figure S16.** Proportion meta-analysis forest plot (random effects) for gestational diabetes.

**Figure S17.** Proportion meta-analysis forest plot (random effects) for elective termination of pregnancy.

**Figure S18.** Proportion meta-analysis forest plot (random effects) for stillbirth.

**Figure S19.** Proportion meta-analysis forest plot (random effects) for caesarean section.

**Figure S20.** Proportion meta-analysis forest plot (random effects) for induction of labour.

**Figure S21.** Proportion meta-analysis forest plot (random effects) for low birth weight by timing of therapy.

**Figure S22.** Proportion meta-analysis forest plot (random effects) for small for gestational age by timing of therapy.

**Figure S23.** Proportion meta-analysis forest plot (random effects) for caesarean section by timing of therapy.

**Figure S24.** Proportion meta-analysis forest plot (random effects) for induction of labour by timing of therapy.

**Appendix F.** Subgroup analyses.

**Table S3.** Subgroup meta-analysis of proportions for preterm birth.

|  | **Number of studies** | **Number of events** | **Number of births** | **Proportion** | **95% CI** | **I^2^ (%)** |
| --- | --- | --- | --- | --- | --- | --- |
| ***Study design*** | | | | | | |
| **Cohort study** | | | | | | |
| Treatment for HL before pregnancy | 7 | 164 | 1,477 | 0.09 | (0.06, 0.14) | 84.96 |
| HL diagnosis during pregnancy | 5 | 153 | 885 | 0.21 | (0.09, 0.36) | 92.34 |
| Never diagnosed with HL | 9 | 995,084 | 12,377,598 | 0.07 | (0.06, 0.08) | 99.96 |
| **Case series** | | | | | | |
| Treatment for HL before pregnancy | 3 | 12 | 220 | 0.04 | (0.00, 0.10) | - |
| HL diagnosis during pregnancy | 7 | 36 | 151 | 0.16 | (0.04, 0.32) | 76.88 |
| ***Location*** | | | | | | |
| **Europe** | | | | | | |
| Treatment for HL before pregnancy | 4 | 28 | 418 | 0.07 | (0.04, 0.09) | 0.00 |
| HL diagnosis during pregnancy | 2 | 12 | 24 | 0.50 | (0.29, 0.71) | - |
| Never diagnosed with HL | 2 | 19,626 | 277,379 | 0.07 | (0.07, 0.07) | - |
| **Americas** | | | | | | |
| Treatment for HL before pregnancy | 6 | 148 | 1,279 | 0.08 | (0.04, 0.14) | 89.24 |
| HL diagnosis during pregnancy | 9 | 132 | 901 | 0.13 | (0.06, 0.20) | 74.24 |
| Never diagnosed with HL | 7 | 975,458 | 12,100,219 | 0.08 | (0.06, 0.09) | 99.97 |
| **Multinational (both Europe and Americas)** | | | | | | |
| HL diagnosis during pregnancy | 1 | 45 | 111 | 0.41 | (0.32, 0.50) | - |
| ***Risk of bias assessment*** | | | | | | |
| **Low** | | | | | | |
| Treatment for HL before pregnancy | 6 | 167 | 1,478 | 0.10 | (0.07, 0.15) | 84.97 |
| HL diagnosis during pregnancy | 5 | 153 | 885 | 0.21 | (0.09, 0.36) | 92.34 |
| Never diagnosed with HL | 7 | 975,964 | 12,109,878 | 0.08 | (0.07, 0.09) | 99.97 |
| **Low-moderate** | | | | | | |
| Treatment for HL before pregnancy | 4 | 9 | 219 | 0.03 | (0.00, 0.08) | 50.10 |
| HL diagnosis during pregnancy | 6 | 32 | 142 | 0.13 | (0.02, 0.29) | 78.70 |
| Never diagnosed with HL | 2 | 19,120 | 267,720 | 0.07 | (0.07, 0.07) | - |
| **Moderate** | | | | | | |
| HL diagnosis during pregnancy | 1 | 4 | 9 | 0.44 | (0.19, 0.73) | - |

**Table S4.** Subgroup meta-analysis of proportions for congenital malformations

|  | **Number of studies** | **Number of events** | **Number of births** | **Proportion** | **95% CI** | **I^2^ (%)** |
| --- | --- | --- | --- | --- | --- | --- |
| ***Study design*** | | | | | | |
| **Cohort study** | | | | | | |
| Treatment for HL before pregnancy | 3 | 22 | 291 | 0.07 | (0.04, 0.10) | 0.00 |
| HL diagnosis during pregnancy | 4 | 9 | 751 | 0.01 | (0.00, 0.05) | 53.71 |
| Never diagnosed with HL | 4 | 31,846 | 7,925,914 | 0.03 | (0.00, 0.07) | 99.57 |
| **Case series** | | | | | | |
| Treatment for HL before pregnancy | 7 | 16 | 384 | 0.03 | (0.00, 0.08) | 60.85 |
| HL diagnosis during pregnancy | 8 | 5 | 132 | 0.01 | (0.00, 0.10) | 61.99 |
| ***Location*** | | | | | | |
| **Europe** | | | | | | |
| Treatment for HL before pregnancy | 5 | 18 | 398 | 0.03 | (0.01, 0.07) | 44.51 |
| HL diagnosis during pregnancy | 2 | 2 | 17 | 0.10 | (0.00, 0.31) | - |
| Never diagnosed with HL | 1 | 341 | 9,279 | 0.04 | (0.03, 0.04) | - |
| **Americas** | | | | | | |
| Treatment for HL before pregnancy | 5 | 20 | 277 | 0.06 | (0.01, 0.13) | 69.51 |
| HL diagnosis during pregnancy | 9 | 10 | 797 | 0.00 | (0.00, 0.04) | 55.98 |
| Never diagnosed with HL | 3 | 31,505 | 7,916,635 | 0.02 | (0.00, 0.15) | - |
| **Multinational (both Europe and Americas)** | | | | | | |
| HL diagnosis during pregnancy | 1 | 2 | 69 | 0.03 | (0.01, 0.10) | - |
| ***Risk of bias assessment*** | | | | | | |
| **Low** | | | | | | |
| Treatment for HL before pregnancy | 2 | 13 | 315 | 0.04 | (0.02, 0.06) | - |
| HL diagnosis during pregnancy | 4 | 9 | 751 | 0.01 | (0.00, 0.05) | 53.71 |
| Never diagnosed with HL | 3 | 31,820 | 7,925,705 | 0.01 | (0.00, 0.05) | 99.52 |
| **Low-moderate** | | | | | | |
| Treatment for HL before pregnancy | 8 | 25 | 360 | 0.06 | (0.02, 0.10) | 53.60 |
| HL diagnosis during pregnancy | 7 | 4 | 128 | 0.01 | (0.00, 0.08) | 60.70 |
| Never diagnosed with HL | 1 | 26 | 209 | 0.12 | (0.09, 0.18) | - |
| **Moderate** | | | | | | |
| HL diagnosis during pregnancy | 1 | 1 | 4 | 0.25 | (0.05, 0.70) | - |

**Table S5.** Subgroup meta-analysis of proportions (random effects) for miscarriage.

|  | **Number of studies** | **Number of events** | **Number of pregnancies** | **Proportion** | **95% CI** | **I^2^ (%)** |
| --- | --- | --- | --- | --- | --- | --- |
| ***Study design*** | | | | | | |
| **Cohort study** | | | | | | |
| Treatment for HL before pregnancy | 4 | 222 | 1547 | 0.13 | (0.10, 0.17) | 47.72 |
| HL diagnosis during pregnancy | 3 | 8 | 769 | 0.03 | (0.00, 0.13) | - |
| Never diagnosed with HL | 3 | 33,255 | 7,918,519 | 0.06 | (0.00, 0.24) | - |
| **Case series** | | | | | | |
| Treatment for HL before pregnancy | 8 | 44 | 477 | 0.07 | (0.03, 0.11) | 45.48 |
| HL diagnosis during pregnancy | 10 | 5 | 166 | 0.01 | (0.00, 0.04) | 11.82 |
| Never diagnosed with HL* | 1 | 11 | 131 | 0.08 | (0.05, 0.14) | 0.00 |
| ***Location*** | | | | | | |
| **Europe** | | | | | | |
| Treatment for HL before pregnancy | 5 | 73 | 591 | 0.10 | (0.06, 0.16) | 50.71 |
| HL diagnosis during pregnancy | 3 | 0 | 23 | 0.00 | (0.00, 0.09) | - |
| **Americas** | | | | | | |
| Treatment for HL before pregnancy | 7 | 193 | 1433 | 0.08 | (0.04, 0.13) | 69.26 |
| HL diagnosis during pregnancy | 9 | 11 | 804 | 0.02 | (0.00, 0.09) | 77.51 |
| Never diagnosed with HL | 4 | 33,266 | 7,918,650 | 0.07 | (0.00, 0.22) | 99.67 |
| **Multinational (both Europe and Americas)** | | | | | | |
| During pregnancy | 1 | 2 | 108 | 0.02 | (0.01, 0.07) | - |
| ***Risk of bias assessment*** | | | | | | |
| **Low** | | | | | | |
| Treatment for HL before pregnancy | 3 | 222 | 1573 | 0.13 | (0.11, 0.16) | - |
| HL diagnosis during pregnancy | 3 | 8 | 769 | 0.03 | (0.00, 0.13) | - |
| Never diagnosed with HL | 2 | 33,235 | 7,918,291 | 0.00 | (0.00, 0.00) | - |
| **Low-moderate** | | | | | | |
| Treatment for HL before pregnancy | 9 | 44 | 451 | 0.07 | (0.03, 0.12) | 53.86 |
| HL diagnosis during pregnancy | 8 | 5 | 151 | 0.01 | (0.00, 0.06) | 31.27 |
| Never diagnosed with HL | 2 | 31 | 359 | 0.09 | (0.06, 0.12) | - |
| **Moderate** | | | | | | |
| HL diagnosis during pregnancy | 2 | 0 | 15 | 0.00 | (0.00, 0.12) | - |

*case series by Aisner et al. included data on miscarriage prior to diagnosis of HL among women included in their study.

**Table S6.** Subgroup meta-analysis of proportions (random effects) for caesarean section.

|  | **Number of studies** | **Number of events** | **Number of births** | **Proportion** | **95% CI** | **I^2^ (%)** |
| --- | --- | --- | --- | --- | --- | --- |
| ***Study design*** | | | | | | |
| **Cohort study** | | | | | | |
| Treatment for HL before pregnancy | 3 | 193 | 625 | 0.29 | (0.20, 0.39) | - |
| HL diagnosis during pregnancy | 3 | 233 | 704 | 0.28 | (0.19, 0.38) | - |
| Never diagnosed with HL | 5 | 2,464,203 | 8,011,619 | 0.26 | (0.22, 0.30) | 99.78 |
| **Case series** | | | | | | |
| Treatment for HL before pregnancy | 2 | 4 | 55 | 0.04 | (0.00, 0.12) | - |
| HL diagnosis during pregnancy | 4 | 10 | 61 | 0.16 | (0.07, 0.27) | 0.00 |
| ***Location*** | | | | | | |
| **Europe** | | | | | | |
| Treatment for HL before pregnancy | 2 | 32 | 164 | 0.17 | (0.11, 0.24) | - |
| HL diagnosis during pregnancy | 1 | 1 | 9 | 0.11 | (0.02, 0.43) | - |
| Never diagnosed with HL | 1 | 5,423 | 25000 | 0.22 | (0.21, 0.22) | - |
| **Americas** | | | | | | |
| Treatment for HL before pregnancy | 3 | 165 | 516 | 0.25 | (0.12, 0.39) | - |
| HL diagnosis during pregnancy | 6 | 242 | 756 | 0.24 | (0.16, 0.33) | 51.08 |
| Never diagnosed with HL | 4 | 2,458,780 | 7986619 | 0.28 | (0.24, 0.32) | 99.62 |
| ***Risk of bias assessment*** | | | | | | |
| **Low** | | | | | | |
| Treatment for HL before pregnancy | 3 | 193 | 625 | 0.29 | (0.20, 0.39) | - |
| HL diagnosis during pregnancy | 3 | 233 | 704 | 0.28 | (0.19, 0.38) | - |
| Never diagnosed with HL | 5 | 2,464,203 | 8011619 | 0.26 | (0.22, 0.30) | 99.78 |
| **Low-moderate** | | | | | | |
| Treatment for HL before pregnancy | 2 | 4 | 55 | 0.04 | (0.00, 0.12) | - |
| HL diagnosis during pregnancy | 3 | 9 | 51 | 0.17 | (0.07, 0.30) | - |
| **Moderate** | | | | | | |
| HL diagnosis during pregnancy | 1 | 1 | 10 | 0.1 | (0.02, 0.40) | - |

**Table S7.** Subgroup meta-analysis of proportions (random effects) for premature rupture of membranes.

|  | **Number of studies** | **Number of events** | **Number of births** | **Proportion** | **95% CI** | **I^2^ (%)** |
| --- | --- | --- | --- | --- | --- | --- |
| ***Study design*** | | | | | | |
| **Cohort study** | | | | | | |
| Treatment for HL before pregnancy | 1 | 17 | 153 | 0.11 | (0.07, 0.17) | - |
| HL diagnosis during pregnancy | 1 | 0 | 638 | 0.00 | (0.00, 0.01) | - |
| Never diagnosed with HL | 2 | 1,939 | 7,941,388 | 0.00 | (0.00, 0.00) | - |
| **Case series** | | | | | | |
| HL diagnosis during pregnancy | 2 | 1 | 57 | 0.01 | (0.00, 0.07) | - |
| ***Location*** | | | | | | |
| **Europe** | | | | | | |
| Treatment for HL before pregnancy | 1 | 17 | 153 | 0.11 | (0.07, 0.17) | - |
| Never diagnosed with HL | 1 | 1,913 | 25,000 | 0.08 | (0.07, 0.08) | - |
| **Americas** | | | | | | |
| HL diagnosis during pregnancy | 3 | 1 | 695 | 0.00 | (0.00, 0.03) | - |
| Never diagnosed with HL | 1 | 26 | 7,916,388 | 0.00 | (0.00, 0.00) | - |
| ***Risk of bias assessment*** | | | | | | |
| **Low** | | | | | | |
| Treatment for HL before pregnancy | 1 | 17 | 153 | 0.11 | (0.07, 0.17) | - |
| HL diagnosis during pregnancy | 1 | 0 | 638 | 0.00 | (0.00, 0.01) | - |
| Never diagnosed with HL | 2 | 1,939 | 7,941,388 | 0.00 | (0.00, 0.00) | - |
| **Low-moderate** | | | | | | |
| HL diagnosis during pregnancy | 2 | 1 | 57 | 0.01 | (0.00, 0.07) | - |

**Table S8.** Subgroup meta-analysis of proportions (random effects) for postpartum haemorrhage.

|  | **Number of studies** | **Number of events** | **Number of births** | **Proportion** | **95% CI** | **I^2^ (%)** |
| --- | --- | --- | --- | --- | --- | --- |
| ***Study design*** | | | | | | |
| **Cohort study** | | | | | | |
| Treatment for HL before pregnancy | 1 | 18 | 153 | 0.12 | (0.08, 0.18) | - |
| HL diagnosis during pregnancy | 1 | 17 | 638 | 0.03 | (0.02, 0.04) | - |
| Never diagnosed with HL | 2 | 173,830 | 7,941,388 | 0.02 | (0.02, 0.02) | - |
| **Case series** | | | | | | |
| HL diagnosis during pregnancy | 1 | 2 | 31 | 0.06 | (0.02, 0.21) | - |
| ***Location*** | | | | | | |
| **Europe** | | | | | | |
| Treatment for HL before pregnancy | 1 | 18 | 153 | 0.12 | (0.08, 0.18) | - |
| Never diagnosed with HL | 1 | 2,179 | 25,000 | 0.09 | (0.08, 0.09) | - |
| **Americas** | | | | | | |
| Treatment for HL before pregnancy | 2 | 19 | 669 | 0.02 | (0.01, 0.04) | - |
| Never diagnosed with HL | 1 | 171,651 | 7,916,388 | 0.02 | (0.02, 0.02) | - |
| ***Risk of bias assessment*** | | | | | | |
| **Low** | | | | | | |
| Treatment for HL before pregnancy | 1 | 18 | 153 | 0.12 | (0.08, 0.18) | - |
| HL diagnosis during pregnancy | 1 | 17 | 638 | 0.03 | (0.02, 0.04) | - |
| Never diagnosed with HL | 2 | 173,830 | 7,941,388 | 0.02 | (0.02, 0.02) | - |
| **Low-moderate** | | | | | | |
| HL diagnosis during pregnancy | 1 | 2 | 31 | 0.06 | (0.02, 0.21) | - |

**Table S9.** Subgroup meta-analysis of proportions (random effects) for anaemia.

|  | **Number of studies** | **Number of events** | **Number of births** | **Proportion** | **95% CI** | **I^2^ (%)** |
| --- | --- | --- | --- | --- | --- | --- |
| ***Study design*** | | | | | | |
| **Cohort study** | | | | | | |
| Treatment for HL before pregnancy | 2 | 14 | 214 | 0.06 | (0.03, 0.10) | - |
| Never diagnosed with HL | 1 | 1,099 | 25,000 | 0.04 | (0.04, 0.05) | - |
| **Case series** | | | | | | |
| HL diagnosis during pregnancy | 2 | 46 | 67 | 0.69 | (0.57, 0.80) | - |
| ***Location*** | | | | | | |
| **Europe** | | | | | | |
| Treatment for HL before pregnancy | 2 | 14 | 214 | 0.06 | (0.03, 0.10) | - |
| Never diagnosed with HL | 1 | 1,099 | 25,000 | 0.04 | (0.04, 0.05) | - |
| **Americas** | | | | | | |
| HL diagnosis during pregnancy | 2 | 46 | 67 | 0.69 | (0.57, 0.80) | - |
| ***Risk of bias assessment*** | | | | | | |
| **Low** | | | | | | |
| Treatment for HL before pregnancy | 2 | 14 | 214 | 0.06 | (0.03, 0.10) | - |
| Never diagnosed with HL | 1 | 1,099 | 25,000 | 0.04 | (0.04, 0.05) | - |
| **Low-moderate** | | | | | | |
| HL diagnosis during pregnancy | 2 | 46 | 67 | 0.69 | (0.57, 0.80) | - |

**Table S10.** Subgroup meta-analysis of proportions (random effects) for 5-minute APGAR score < 7.

|  | **Number of studies** | **Number of events** | **Number of births** | **Proportion** | **95% CI** | **I^2^ (%)** |
| --- | --- | --- | --- | --- | --- | --- |
| ***Study design*** | | | | | | |
| **Cohort study** | | | | | | |
| Treatment for HL before pregnancy | 2 | 6 | 472 | 0.01 | (0.00, 0.03) | - |
| HL diagnosis during pregnancy | 1 | 8 | 93 | 0.09 | (0.04, 0.16) | - |
| Never diagnosed with HL | 2 | 5,933 | 151,616 | 0.04 | (0.04, 0.04) | - |
| **Case series** | | | | | | |
| HL diagnosis during pregnancy | 1 | 0 | 9 | 0.00 | (0.00, 0.30) | - |
| ***Location*** | | | | | | |
| **Europe** | | | | | | |
| HL diagnosis during pregnancy | 1 | 0 | 9 | 0.00 | (0.00, 0.30) | - |
| **Americas** | | | | | | |
| Treatment for HL before pregnancy | 2 | 6 | 472 | 0.01 | (0.00, 0.03) | - |
| HL diagnosis during pregnancy | 1 | 8 | 93 | 0.09 | (0.04, 0.16) | - |
| Never diagnosed with HL | 2 | 5,933 | 151,616 | 0.04 | (0.04, 0.04) | - |
| ***Risk of bias assessment*** | | | | | | |
| **Low** | | | | | | |
| Treatment for HL before pregnancy | 2 | 6 | 472 | 0.01 | (0.00, 0.03) | - |
| HL diagnosis during pregnancy | 1 | 8 | 93 | 0.09 | (0.04, 0.16) | - |
| Never diagnosed with HL | 2 | 5,933 | 151,616 | 0.04 | (0.04, 0.04) | - |
| **Moderate** | | | | | | |
| HL diagnosis during pregnancy | 1 | 0 | 9 | 0.00 | (0.00, 0.30) | - |

**Table S11.** Subgroup meta-analysis of proportions (random effects) for low birth weight.

|  | **Number of studies** | **Number of events** | **Number of births** | **Proportion** | **95% CI** | **I^2^ (%)** |
| --- | --- | --- | --- | --- | --- | --- |
| ***Study design*** | | | | | | |
| **Cohort study** | | | | | | |
| Treatment for HL before pregnancy | 7 | 79 | 1,396 | 0.05 | (0.03, 0.08) | 73.20 |
| HL diagnosis during pregnancy | 3 | 41 | 212 | 0.13 | (0.01, 0.32) | - |
| Never diagnosed with HL | 6 | 815,251 | 12,058,985 | 0.05 | (0.05, 0.06) | 99.77 |
| **Case series** | | | | | | |
| Treatment for HL before pregnancy | 2 | 3 | 145 | 0.01 | (0.00, 0.04) | - |
| HL diagnosis during pregnancy | 6 | 8 | 84 | 0.06 | (0.00, 0.16) | 19.67 |
| ***Location*** | | | | | | |
| **Europe** | | | | | | |
| Treatment for HL before pregnancy | 5 | 23 | 717 | 0.02 | (0.01, 0.05) | 52.11 |
| HL diagnosis during pregnancy | 4 | 4 | 29 | 0.11 | (0.00, 0.31) | 27.19 |
| Never diagnosed with HL | 2 | 529,143 | 7,874,769 | 0.07 | (0.07, 0.07) | - |
| **Americas** | | | | | | |
| Treatment for HL before pregnancy | 4 | 59 | 824 | 0.07 | (0.05, 0.09) | 26.64 |
| HL diagnosis during pregnancy | 4 | 13 | 156 | 0.06 | (0.03, 0.12) | 0.00 |
| Never diagnosed with HL | 4 | 286,108 | 4,184,216 | 0.06 | (0.04, 0.07) | 99.79 |
| **Multinational (both Europe and Americas)** | | | | | | |
| HL diagnosis during pregnancy | 1 | 32 | 111 | 0.29 | (0.21, 0.38) | - |
| ***Risk of bias assessment*** | | | | | | |
| **Low** | | | | | | |
| Treatment for HL before pregnancy | 6 | 75 | 1,466 | 0.05 | (0.03, 0.07) | 76.04 |
| HL diagnosis during pregnancy | 3 | 41 | 212 | 0.13 | (0.01, 0.32) | - |
| Never diagnosed with HL | 5 | 286,262 | 4,193,539 | 0.05 | (0.03, 0.06) | 99.81 |
| **Low-moderate** | | | | | | |
| Treatment for HL before pregnancy | 3 | 7 | 75 | 0.08 | (0.02, 0.16) | - |
| HL diagnosis during pregnancy | 4 | 5 | 71 | 0.05 | (0.00, 0.13) | 0.00 |
| Never diagnosed with HL | 1 | 528,989 | 7,865,446 | 0.07 | (0.07, 0.07) | - |
| **Moderate** |  |  |  |  |  |  |
| HL diagnosis during pregnancy | 2 | 3 | 13 | 0.20 | (0.01, 0.49) | - |

**Table S12.** Subgroup meta-analysis of proportions (random effects) for small for gestational age.

|  | **Number of studies** | **Number of events** | **Number of births** | **Proportion** | **95% CI** | **I^2^ (%)** |
| --- | --- | --- | --- | --- | --- | --- |
| ***Study design*** | | | | | | |
| **Cohort study** | | | | | | |
| Treatment for HL before pregnancy | 3 | 78 | 809 | 0.10 | (0.08, 0.12) | - |
| HL diagnosis during pregnancy | 3 | 47 | 837 | 0.10 | (0.01, 0.25) | - |
| Never diagnosed with HL | 5 | 605,552 | 12,089,330 | 0.08 | (0.03, 0.15) | 100.00 |
| **Case series** | | | | | | |
| HL diagnosis during pregnancy | 3 | 8 | 42 | 0.17 | (0.05, 0.33) | - |
| ***Location*** | | | | | | |
| **Europe** | | | | | | |
| HL diagnosis during pregnancy | 1 | 2 | 4 | 0.50 | (0.15, 0.85) | - |
| **Americas** | | | | | | |
| Treatment for HL before pregnancy | 3 | 78 | 809 | 0.10 | (0.08, 0.12) | - |
| HL diagnosis during pregnancy | 5 | 53 | 875 | 0.11 | (0.03, 0.24) | 91.86 |
| Never diagnosed with HL | 5 | 605,552 | 12,089,330 | 0.08 | (0.03, 0.15) | 100.00 |
| ***Risk of bias assessment*** | | | | | | |
| **Low** | | | | | | |
| Treatment for HL before pregnancy | 3 | 78 | 809 | 0.10 | (0.08, 0.12) | - |
| HL diagnosis during pregnancy | 3 | 47 | 837 | 0.10 | (0.01, 0.25) | - |
| Never diagnosed with HL | 5 | 605,552 | 12,089,330 | 0.08 | (0.03, 0.15) | 100.00 |
| **Low-moderate** | | | | | | |
| HL diagnosis during pregnancy | 1 | 4 | 27 | 0.15 | (0.06, 0.32) | - |
| **Moderate** | | | | | | |
| HL diagnosis during pregnancy | 1 | 2 | 4 | 0.50 | (0.15, 0.85) | - |
| **High** | | | | | | |
| HL diagnosis during pregnancy | 1 | 2 | 11 | 0.18 | (0.05, 0.48) | - |

**Table S13.** Subgroup meta-analysis of proportions (random effects) for neonatal death.

|  | **Number of studies** | **Number of events** | **Number of births** | **Proportion** | **95% CI** | **I^2^ (%)** |
| --- | --- | --- | --- | --- | --- | --- |
| ***Study design*** | | | | | | |
| **Cohort study** | | | | | | |
| Treatment for HL before pregnancy | 2 | 0 | 97 | 0.00 | (0.00, 0.01) | - |
| HL diagnosis during pregnancy | 1 | 1 | 131 | 0.01 | (0.00, 0.04) | - |
| Never diagnosed with HL | 2 | 2 | 244 | 0.01 | (0.00, 0.02) | - |
| **Case series** | | | | | | |
| Treatment for HL before pregnancy | 1 | 0 | 12 | 0.00 | (0.00, 0.24) | - |
| HL diagnosis during pregnancy | 2 | 0 | 48 | 0.00 | (0.00, 0.01) | - |
| ***Location*** | | | | | | |
| **Europe** | | | | | | |
| HL diagnosis during pregnancy | 1 | 0 | 4 | 0.00 | (0.00, 0.49) | - |
| **Americas** | | | | | | |
| Treatment for HL before pregnancy | 3 | 0 | 109 | 0.00 | (0.00, 0.01) | - |
| HL diagnosis during pregnancy | 2 | 1 | 175 | 0.01 | (0.00, 0.02) | - |
| Never diagnosed with HL | 2 | 2 | 244 | 0.01 | (0.00, 0.02) | - |
| ***Risk of bias assessment*** | | | | | | |
| **Low** | | | | | | |
| HL diagnosis during pregnancy | 1 | 1 | 131 | 0.01 | (0.00, 0.04) | - |
| Never diagnosed with HL | 1 | 0 | 38 | 0.00 | (0.00, 0.09) | - |
| **Low-moderate** | | | | | | |
| Treatment for HL before pregnancy | 3 | 0 | 109 | 0.00 | (0.00, 0.01) | - |
| HL diagnosis during pregnancy | 1 | 0 | 44 | 0.00 | (0.00, 0.08) | - |
| Never diagnosed with HL | 1 | 2 | 206 | 0.01 | (0.00, 0.03) | - |
| **Moderate** | | | | | | |
| HL diagnosis during pregnancy | 1 | 0 | 4 | 0.00 | (0.00, 0.49) | - |

**Table S14.** Subgroup meta-analysis of proportions (random effects) for pregnancy-induced hypertension.

|  | **Number of studies** | **Number of events** | **Number of births** | **Proportion** | **95% CI** | **I^2^ (%)** |
| --- | --- | --- | --- | --- | --- | --- |
| ***Study design*** | | | | | | |
| **Cohort study** | | | | | | |
| Treatment for HL before pregnancy | 1 | 6 | 153 | 0.04 | (0.02, 0.08) | - |
| HL diagnosis during pregnancy | 1 | 14 | 638 | 0.02 | (0.01, 0.04) | - |
| Never diagnosed with HL | 2 | 247,921 | 7,941,388 | 0.03 | (0.03, 0.03) | - |
| ***Location*** | | | | | | |
| **Europe** | | | | | | |
| Treatment for HL before pregnancy | 1 | 6 | 153 | 0.04 | (0.02, 0.08) | - |
| Never diagnosed with HL | 1 | 1,013 | 25000 | 0.04 | (0.04, 0.04) | - |
| **Americas** | | | | | | |
| HL diagnosis during pregnancy | 1 | 14 | 638 | 0.02 | (0.01, 0.04) | - |
| Never diagnosed with HL | 1 | 246,908 | 7,916,388 | 0.03 | (0.03, 0.03) | - |
| ***Risk of bias assessment*** | | | | | | |
| **Low** | | | | | | |
| Treatment for HL before pregnancy | 1 | 6 | 153 | 0.04 | (0.02, 0.08) | - |
| HL diagnosis during pregnancy | 1 | 14 | 638 | 0.02 | (0.01, 0.04) | - |
| Never diagnosed with HL | 2 | 247,921 | 7,941,388 | 0.03 | (0.03, 0.03) | - |

**Table S15.** Subgroup meta-analysis of proportions (random effects) for pre-eclampsia.

|  | **Number of studies** | **Number of events** | **Number of births** | **Proportion** | **95% CI** | **I^2^ (%)** |
| --- | --- | --- | --- | --- | --- | --- |
| ***Study design*** | | | | | | |
| **Cohort study** | | | | | | |
| Treatment for HL before pregnancy | 1 | 4 | 61 | 0.07 | (0.03, 0.16) | - |
| HL diagnosis during pregnancy | 2 | 40 | 732 | 0.05 | (0.04, 0.07) | - |
| Never diagnosed with HL | 2 | 316,856 | 8,055,486 | 0.04 | (0.04, 0.04) | - |
| **Case series** | | | | | | |
| HL diagnosis during pregnancy | 2 | 1 | 41 | 0.00 | (0.00, 0.07) | - |
| ***Location*** | | | | | | |
| **Europe** | | | | | | |
| Treatment for HL before pregnancy | 1 | 4 | 61 | 0.07 | (0.03, 0.16) | - |
| **Americas** | | | | | | |
| HL diagnosis during pregnancy | 4 | 41 | 773 | 0.04 | (0.01, 0.07) | 32.78 |
| Never diagnosed with HL | 2 | 316,856 | 8,055,486 | 0.04 | (0.04, 0.04) | - |
| ***Risk of bias assessment*** | | | | | | |
| **Low** | | | | | | |
| Treatment for HL before pregnancy | 1 | 4 | 61 | 0.07 | (0.03, 0.16) | - |
| HL diagnosis during pregnancy | 2 | 40 | 732 | 0.05 | (0.04, 0.07) | - |
| Never diagnosed with HL | 2 | 316,856 | 8,055,486 | 0.04 | (0.04, 0.04) | - |
| **Low-moderate** | | | | | | |
| HL diagnosis during pregnancy | 2 | 1 | 41 | 0.00 | (0.00, 0.07) | - |

**Table S16.** Subgroup meta-analysis of proportions (random effects) for gestational diabetes.

|  | **Number of studies** | **Number of events** | **Number of births** | **Proportion** | **95% CI** | **I^2^ (%)** |
| --- | --- | --- | --- | --- | --- | --- |
| ***Study design*** | | | | | | |
| **Cohort study** | | | | | | |
| Treatment for HL before pregnancy | 1 | 5 | 153 | 0.03 | (0.01, 0.07) | - |
| HL diagnosis during pregnancy | 2 | 48 | 732 | 0.06 | (0.05, 0.08) | - |
| Never diagnosed with HL | 3 | 429,742 | 8,080,486 | 0.04 | (0.03, 0.06) | - |
| **Case series** | | | | | | |
| HL diagnosis during pregnancy | 1 | 1 | 31 | 0.03 | (0.01, 0.16) | - |
| ***Location*** | | | | | | |
| **Europe** | | | | | | |
| Treatment for HL before pregnancy | 1 | 5 | 153 | 0.03 | (0.01, 0.07) | - |
| Never diagnosed with HL | 1 | 390 | 25,000 | 0.02 | (0.01, 0.02) | - |
| **Americas** | | | | | | |
| HL diagnosis during pregnancy | 3 | 49 | 763 | 0.06 | (0.04, 0.08) | - |
| Never diagnosed with HL | 2 | 429,352 | 8,055,486 | 0.05 | (0.05, 0.05) | - |
| ***Risk of bias assessment*** | | | | | | |
| **Low** | | | | | | |
| Treatment for HL before pregnancy | 1 | 5 | 153 | 0.03 | (0.01, 0.07) | - |
| HL diagnosis during pregnancy | 2 | 48 | 732 | 0.06 | (0.05, 0.08) | - |
| Never diagnosed with HL | 3 | 429,742 | 8,080,486 | 0.04 | (0.03, 0.06) | - |
| **Low-moderate** | | | | | | |
| HL diagnosis during pregnancy | 1 | 1 | 31 | 0.03 | (0.01, 0.16) | - |

**Table S17.** Subgroup meta-analysis of proportions (random effects) for elective termination of pregnancy.

|  | **Number of studies** | **Number of events** | **Number of pregnancies** | **Proportion** | **95% CI** | **I^2^ (%)** |
| --- | --- | --- | --- | --- | --- | --- |
| ***Study design*** | | | | | | |
| **Cohort study** | | | | | | |
| Treatment for HL before pregnancy | 4 | 214 | 1,547 | 0.11 | (0.06, 0.16) | 80.51 |
| HL diagnosis during pregnancy | 2 | 14 | 184 | 0.07 | (0.04, 0.12) | - |
| Never diagnosed with HL | 2 | 220 | 1,996 | 0.10 | (0.09, 0.12) | - |
| **Case series** | | | | | | |
| Treatment for HL before pregnancy | 7 | 50 | 332 | 0.14 | (0.10, 0.19) | 11.78 |
| During pregnancy | 11 | 31 | 222 | 0.13 | (0.04, 0.24) | 74.83 |
| Never diagnosed with HL* | 1 | 13 | 131 | 0.10 | (0.06, 0.16) | - |
| ***Location*** | | | | | | |
| **Europe** | | | | | | |
| Treatment for HL before pregnancy | 4 | 59 | 446 | 0.12 | (0.07, 0.19) | 34.60 |
| HL diagnosis during pregnancy | 3 | 1 | 24 | 0.02 | (0.00, 0.14) | - |
| **Americas** | | | | | | |
| Treatment for HL before pregnancy | 7 | 205 | 1,433 | 0.12 | (0.07, 0.17) | 66.53 |
| HL diagnosis during pregnancy | 9 | 34 | 248 | 0.15 | (0.06, 0.27) | 78.42 |
| Never diagnosed with HL | 3 | 233 | 2,127 | 0.06 | (0.01, 0.15) | - |
| **Multinational (both Europe and Americas)** | | | | | | |
| HL diagnosis during pregnancy | 1 | 10 | 134 | 0.07 | (0.04, 0.13) | - |
| ***Risk of bias assessment*** | | | | | | |
| **Low** | | | | | | |
| Treatment for HL before pregnancy | 2 | 207 | 1,428 | 0.14 | (0.13, 0.16) | - |
| HL diagnosis during pregnancy | 2 | 14 | 184 | 0.07 | (0.04, 0.12) | - |
| Never diagnosed with HL | 1 | 220 | 1,903 | 0.12 | (0.10, 0.13) | - |
| **Low-moderate** | | | | | | |
| Treatment for HL before pregnancy | 9 | 57 | 451 | 0.12 | (0.07, 0.18) | 59.83 |
| HL diagnosis during pregnancy | 9 | 30 | 207 | 0.14 | (0.04, 0.27) | 78.89 |
| Never diagnosed with HL | 2 | 13 | 224 | 0.04 | (0.02, 0.07) | - |
| **Moderate** | | | | | | |
| HL diagnosis during pregnancy | 2 | 1 | 15 | 0.03 | (0.00, 0.23) | - |

*case series by Aisner et al. included data on elective termination prior to diagnosis of HL among women included in their study.

**Table S18.** Subgroup meta-analysis of proportions (random effects) for stillbirth.

|  | **Number of studies** | **Number of events** | **Number of births** | **Proportion** | **95% CI** | **I^2^ (%)** |
| --- | --- | --- | --- | --- | --- | --- |
| ***Study design*** | | | | | | |
| **Cohort study** | | | | | | |
| Treatment for HL before pregnancy | 5 | 17 | 1,728 | 0.01 | (0.00, 0.01) | 0.00 |
| HL diagnosis during pregnancy | 3 | 4 | 189 | 0.01 | (0.00, 0.04) | - |
| Never diagnosed with HL | 5 | 89,688 | 13,066,864 | 0.00 | (0.00, 0.01) | 80.29 |
| **Case series** | | | | | | |
| Treatment for HL before pregnancy | 5 | 4 | 257 | 0.01 | (0.00, 0.04) | 46.55 |
| HL diagnosis during pregnancy | 5 | 1 | 77 | 0.00 | (0.00, 0.02) | 0.00 |
| Never diagnosed with HL* | 1 | 1 | 131 | 0.01 | (0.00, 0.04) | - |
| ***Location*** | | | | | | |
| **Europe** | | | | | | |
| Treatment for HL before pregnancy | 5 | 4 | 707 | 0.00 | (0.00, 0.00) | 0.00 |
| HL diagnosis during pregnancy | 3 | 1 | 29 | 0.01 | (0.00, 0.14) | - |
| Never diagnosed with HL | 2 | 89,672 | 13,064,716 | 0.01 | (0.01, 0.01) | - |
| **Americas** | | | | | | |
| Treatment for HL before pregnancy | 5 | 17 | 1278 | 0.01 | (0.01, 0.03) | 38.25 |
| HL diagnosis during pregnancy | 4 | 2 | 103 | 0.00 | (0.00, 0.04) | 0.00 |
| Never diagnosed with HL | 4 | 17 | 2279 | 0.00 | (0.00, 0.01) | 0.00 |
| **Multinational (both Europe and Americas)** | | | | | | |
| HL diagnosis during pregnancy | 1 | 2 | 134 | 0.01 | (0.00, 0.05) | - |
| ***Risk of bias assessment*** | | | | | | |
| **Low** | | | | | | |
| Treatment for HL before pregnancy | 4 | 15 | 1752 | 0.01 | (0.00. 0.01) | 0.00 |
| HL diagnosis during pregnancy | 3 | 4 | 189 | 0.01 | (0.00, 0.04) | - |
| Never diagnosed with HL | 3 | 50 | 11894 | 0.00 | (0.00, 0.00) | - |
| **Low-moderate** | | | | | | |
| Treatment for HL before pregnancy | 6 | 6 | 233 | 0.02 | (0.00, 0.04) | 0.00 |
| HL diagnosis during pregnancy | 4 | 0 | 72 | 0.00 | (0.00, 0.02) | 0.00 |
| Never diagnosed with HL | 3 | 89,639 | 13,055,101 | 0.01 | (0.00, 0.01) | - |
| **Moderate** | | | | | | |
| HL diagnosis during pregnancy | 1 | 1 | 5 | 0.20 | (0.04, 0.62) | 0.00 |

*case series by Aisner et al. included data on stillbirth prior to diagnosis of HL among women included in their study.

**Table S19.** Subgroup meta-analysis of proportions (random effects) for induction of labour.

|  | **Number of studies** | **Number of events** | **Number of births** | **Proportion** | **95% CI** | **I^2^ (%)** |
| --- | --- | --- | --- | --- | --- | --- |
| ***Study design*** | | | | | | |
| **Cohort study** | | | | | | |
| HL diagnosis during pregnancy | 1 | 10 | 638 | 0.02 | (0.01, 0.03) | - |
| Never diagnosed with HL | 1 | 97,054 | 7,916,388 | 0.01 | (0.01, 0.01) | - |
| **Case series** | | | | | | |
| HL diagnosis during pregnancy | 4 | 14 | 58 | 0.15 | (0.01, 0.38) | 67.20 |
| ***Location*** | | | | | | |
| **Europe** | | | | | | |
| HL diagnosis during pregnancy | 1 | 0 | 9 | 0.00 | (0.00, 0.30) | - |
| **Americas** | | | | | | |
| HL diagnosis during pregnancy | 4 | 24 | 687 | 0.12 | (0.00, 0.43) | 92.52 |
| Never diagnosed with HL | 1 | 97,054 | 7,916,388 | 0.01 | (0.01, 0.01) | - |
| ***Risk of bias assessment*** | | | | | | |
| **Low** | | | | | | |
| HL diagnosis during pregnancy | 1 | 10 | 638 | 0.02 | (0.01, 0.03) | - |
| Never diagnosed with HL | 1 | 97,054 | 7,916,388 | 0.01 | (0.01, 0.01) | - |
| **Low-moderate** | | | | | | |
| During pregnancy | 4 | 14 | 58 | 0.15 | (0.01, 0.38) | 67.20 |

**Appendix G:** Sensitivity analyses.

The studies by El-Messidi et al.^53^ and Dilek et al.^52^ reported data on IUGR rather than SGA. While not all babies delivered SGA are growth restricted, the majority of babies with IUGR are delivered SGA.^82^ The data from these studies, therefore, were included in the meta-analysis for SGA. Also, data provided by BA Mueller regarding the publication by Niu et al.^38^ in personal communication^73^ contained pre-eclampsia and eclampsia data grouped together. For Swerdlow et al.,^69^ outcome data included females and female partners of males grouped together, although it was noted that proportions of both were similar.

Kroll-Balcerzak et al.^71^ and McKeen et al.^72^ reported outcomes in the form of a clinical vignette and letter to the editor respectively. These forms of publications contain less information than full papers, and letters to the editor are often not peer-reviewed, as was the case for McKeen et al. Women diagnosed with HL after 24 weeks gestation are no longer at risk of miscarriage as loss of pregnancy after this time is defined as stillbirth,^30^ therefore, where trimester or gestation at diagnosis were reported, those patients diagnosed after 24 weeks or during the third trimester were excluded from analysis of miscarriage. Five studies did not report on gestation or trimester at diagnosis,^48,52,63,70,72^ and the inclusion of these studies may have resulted in the underestimation of the proportion of miscarriage. Sensitivity analyses were performed with data from the above-mentioned studies excluded and results compared to the overall meta-analyses (Table S18).

Of the 33 studies included in this systematic review, eight were published before 1990 and 15 before 2000. Meta-analyses of congenital malformations, PTB, miscarriage, CS, LBW, neonatal death, pre-eclampsia, elective termination, stillbirth and IOL were repeated with studies published before 1990 and 2000 excluded separately and results compared to the original meta-analyses for these outcomes (Table S19).

**Table S20.** Sensitivity meta-analysis of proportions (random effects) with studies containing heterogeneous outcome data excluded.

|  | **Number of studies** | **Number of events** | **Number of births/pregnancies** | **Proportion** | **95% CI** | **I^2^ (%)** |
| --- | --- | --- | --- | --- | --- | --- |
| **Congenital malformations excluding studies by Swerdlow et al. and McKeen et al.** | | | | | | |
| Treatment for HL before pregnancy | 8 | 30 | 613 | 0.03 | (0.01, 0.07) | 55.64 |
| Treatment for HL during pregnancy | 11 | 10 | 875 | 0.00 | (0.00, 0.00) | 8.15 |
| Never diagnosed with HL | 4 | 31,846 | 7,925,914 | 0.03 | (0.00, 0.07) | 99.57 |
| **Preterm birth excluding studies by Swerdlow et al., Kroll-Balcerzak et al. and McKeen et al.** | | | | | | |
| Treatment for HL before pregnancy | 9 | 172 | 1,648 | 0.08 | (0.05, 0.12) | 84.84 |
| Treatment for HL during pregnancy | 10 | 185 | 1,019 | 0.19 | (0.10, 0.29) | 87.96 |
| Never diagnosed with HL | 8 | 975,967 | 12,110,085 | 0.07 | (0.06, 0.09) | 99.97 |
| **Miscarriage excluding studies by Swerdlow et al., Kroll-Balcerzak et al. and McKeen et al.** | | | | | | |
| Treatment for HL before pregnancy | 10 | 256 | 1,954 | 0.09 | (0.06, 0.12) | 64.59 |
| Treatment for HL during pregnancy | 11 | 12 | 912 | 0.01 | (0.00, 0.05) | 71.23 |
| Never diagnosed with HL | 4 | 33,266 | 7,918,650 | 0.07 | (0.00, 0.22) | 99.67 |
| **Miscarriage excluding studies which did not report trimester at diagnosis.** | | | | | | |
| HL diagnosis during pregnancy | 8 | 6 | 200 | 0.00 | (0.00, 0.03) | 3.67 |
| Never diagnosed with HL | 3 | 310 | 2,262 | 0.11 | (0.07, 0.16) | - |
| **5-minute APGAR score < 7 excluding study by Kroll-Balcerzak et al.** | | | | | | |
| Treatment for HL during pregnancy | 1 | 8 | 93 | 0.09 | (0.04, 0.16) | - |
| Never diagnosed with HL | 2 | 5,933 | 151,616 | 0.04 | (0.04, 0.04) | - |
| **Low birth weight excluding studies by Swerdlow et al., Kroll-Balcerzak et al. and McKeen et al.** | | | | | | |
| Treatment for HL before pregnancy | 8 | 77 | 1,492 | 0.04 | (0.02, 0.07) | 69.75 |
| Treatment for HL during pregnancy | 7 | 48 | 279 | 0.11 | (0.03, 0.22) | 73.23 |
| Never diagnosed with HL | 5 | 286,262 | 4,193,539 | 0.05 | (0.03, 0.06) | 99.81 |
| **Small for gestational age excluding studies by El-Messidi et al. and Dilek et al.** | | | | | | |
| HL diagnosis during pregnancy | 4 | 38 | 237 | 0.15 | (0.11, 0.20) | 0.00 |
| Never diagnosed with HL | 4 | 447,336 | 4,172,942 | 0.10 | (0.09, 0.11) | 99.31 |
| **Pre-eclampsia excluding data provided by BA Mueller (Niu et al.)** | | | | | | |
| HL diagnosis during pregnancy | 3 | 34 | 679 | 0.03 | (0.00, 0.08) | - |
| Never diagnosed with HL | 1 | 308,520 | 7,916,388 | 0.04 | (0.04, 0.04) | - |
| **Elective termination excluding studies by Swerdlow et al., Kroll-Balcerzak et al. and McKeen et al.** | | | | | | |
| HL diagnosis before pregnancy | 9 | 256 | 1,809 | 0.12 | (0.09, 0.16) | 63.50 |
| HL diagnosis during pregnancy | 11 | 40 | 383 | 0.10 | (0.04, 0.18) | 70.90 |
| Never diagnosed with HL | 3 | 233 | 2,127 | 0.06 | (0.01, 0.15) | - |
| **Stillbirth excluding studies by Swerdlow et al. and McKeen et al.** | | | | | | |
| Treatment for HL before pregnancy | 8 | 19 | 1,915 | 0.00 | (0.00, 0.01) | 0.00 |
| Treatment for HL during pregnancy | 7 | 5 | 258 | 0.00 | (0.00, 0.02) | 0.00 |
| Never diagnosed with HL | 5 | 54 | 12,232 | 0.00 | (0.00, 0.01) | 47.22 |

**Table S21.** Sensitivity meta-analysis of proportions (random effects) with studies published prior to 2000 and 1990 excluded.

|  | **Number of studies** | **Number of events** | **Number of pregnancies/births** | **Proportion** | **95% CI** | **I^2^ (%)** |
| --- | --- | --- | --- | --- | --- | --- |
| ***Congenital malformations*** | | | | | | |
| **Studies published prior to 2000 excluded** | | | | | | |
| Treatment for HL before pregnancy | 2 | 13 | 315 | 0.04 | (0.02, 0.06) | - |
| HL diagnosis during pregnancy | 6 | 9 | 799 | 0.00 | (0.00, 0.02) | 45.71 |
| Never diagnosed with HL | 2 | 31,820 | 7,925,667 | 0.00 | (0.00, 0.00) | - |
| **Studies published prior to 1990 excluded** | | | | | | |
| Treatment for HL before pregnancy | 6 | 23 | 501 | 0.04 | (0.01, 0.08) | 65.15 |
| HL diagnosis during pregnancy | 8 | 10 | 846 | 0.00 | (0.00, 0.02) | 34.24 |
| Never diagnosed with HL | 3 | 31,820 | 7,925,705 | 0.01 | (0.00, 0.05) | - |
| ***Preterm birth*** | | | | | | |
| **Studies published prior to 2000 excluded** | | | | | | |
| Treatment for HL before pregnancy | 6 | 167 | 1,478 | 0.10 | (0.07, 0.15) | 84.97 |
| HL diagnosis during pregnancy | 8 | 188 | 972 | 0.29 | (0.18, 0.41) | 88.72 |
| Never diagnosed with HL | 6 | 975,963 | 12,109,841 | 0.08 | (0.07, 0.09) | 99.98 |
| **Studies published prior to 1990 excluded** | | | | | | |
| Treatment for HL before pregnancy | 8 | 171 | 1,569 | 0.09 | (0.05, 0.13) | 84.35 |
| HL diagnosis during pregnancy | 10 | 189 | 1,017 | 0.22 | (0.13, 0.33) | 87.91 |
| Never diagnosed with HL | 8 | 995,081 | 12,377,391 | 0.08 | (0.07, 0.09) | 99.97 |
| ***Miscarriage*** | | | | | | |
| **Studies published prior to 2000 excluded** | | | | | | |
| Treatment for HL before pregnancy | 3 | 222 | 1,573 | 0.13 | (0.11, 0.16) | - |
| HL diagnosis during pregnancy | 7 | 7 | 893 | 0.00 | (0.00, 0.03) | 64.56 |
| Never diagnosed with HL | 2 | 33,235 | 7,918,291 | 0.00 | (0.00, 0.00) | - |
| **Studies published prior to 1990 excluded** | | | | | | |
| Treatment for HL before pregnancy | 7 | 249 | 1,798 | 0.11 | (0.08, 0.15) | 68.11 |
| HL diagnosis during pregnancy | 10 | 12 | 968 | 0.01 | (0.00, 0.04) | 69.70 |
| Never diagnosed with HL | 3 | 33,246 | 7,918,422 | 0.06 | (0.00, 0.24) | - |
| ***Caesarean section*** | | | | | | |
| **Studies published prior to 2000 excluded** | | | | | | |
| Treatment for HL before pregnancy | 3 | 193 | 625 | 0.29 | (0.20, 0.39) | - |
| HL diagnosis during pregnancy | 3 | 234 | 710 | 0.27 | (0.18, 0.38) | - |
| Never diagnosed with HL | 4 | 2,464,197 | 8,011,581 | 0.27 | (0.23, 0.31) | 99.84 |
| **Studies published prior to 1990 excluded** | | | | | | |
| Treatment for HL before pregnancy | 5 | 197 | 680 | 0.22 | (0.12, 0.33) | 87.17 |
| HL diagnosis during pregnancy | 5 | 240 | 744 | 0.25 | (0.16, 0.34) | 55.05 |
| Never diagnosed with HL | 5 | 2,464,203 | 8,011,619 | 0.26 | (0.22, 0.30) | 99.78 |
| ***Low birth weight*** | | | | | | |
| **Studies published prior to 2000 excluded** | | | | | | |
| Treatment for HL before pregnancy | 6 | 75 | 1,466 | 0.05 | (0.03, 0.07) | 76.04 |
| HL diagnosis during pregnancy | 6 | 48 | 269 | 0.13 | (0.04, 0.26) | 74.14 |
| Never diagnosed with HL | 5 | 286,262 | 4,193,539 | 0.05 | (0.03, 0.06) | 99.81 |
| **Studies published prior to 1990 excluded** | | | | | | |
| Treatment for HL before pregnancy | 9 | 82 | 1,541 | 0.04 | (0.02, 0.07) | 68.66 |
| HL diagnosis during pregnancy | 8 | 49 | 288 | 0.11 | (0.03, 0.21) | 68.80 |
| Never diagnosed with HL | 6 | 815,251 | 12,058,985 | 0.05 | (0.05, 0.06) | 99.77 |
| ***Neonatal death*** | | | | | | |
| **Studies published prior to 2000 excluded** | | | | | | |
| Treatment for HL before pregnancy | 0 | - | - | - | - | - |
| HL diagnosis during pregnancy | 2 | 0 | 48 | 0.00 | (0.00, 0.01) | - |
| Never diagnosed with HL | 0 | - | - | - | - | - |
| **Studies published prior to 1990 excluded** | | | | | | |
| Treatment for HL before pregnancy | 1 | 0 | 15 | 0.00 | (0.00, 0.20) | - |
| HL diagnosis during pregnancy | 3 | 1 | 179 | 0.00 | (0.00, 0.00) | - |
| Never diagnosed with HL | 1 | 0 | 38 | 0.00 | (0.00, 0.09) | - |
| ***Pre-eclampsia*** | | | | | | |
| **Studies published prior to 2000 excluded** | | | | | | |
| Treatment for HL before pregnancy | 1 | 4 | 61 | 0.07 | (0.03, 0.16) | - |
| HL diagnosis during pregnancy | 3 | 40 | 763 | 0.04 | (0.02, 0.08) | - |
| Never diagnosed with HL | 2 | 316,856 | 8,055,486 | 0.04 | (0.04, 0.04) | - |
| **Studies published prior to 1990 excluded** | | | | | | |
| Treatment for HL before pregnancy | 1 | 4 | 61 | 0.07 | (0.03, 0.16) | - |
| HL diagnosis during pregnancy | 3 | 40 | 763 | 0.04 | (0.02, 0.08) | - |
| Never diagnosed with HL | 2 | 316,856 | 8,055,486 | 0.04 | (0.04, 0.04) | - |
| ***Elective termination*** | | | | | | |
| **Studies published prior to 2000 excluded** | | | | | | |
| Treatment for HL before pregnancy | 2 | 207 | 1,428 | 0.14 | (0.13, 0.16) | - |
| HL diagnosis during pregnancy | 6 | 16 | 264 | 0.03 | (0.00, 0.08) | 38.49 |
| Never diagnosed with HL | 1 | 220 | 1,903 | 0.12 | (0.10, 0.13) | - |
| **Studies published prior to 1990 excluded** | | | | | | |
| Treatment for HL before pregnancy | 6 | 239 | 1,653 | 0.14 | (0.12, 0.15) | 0.00 |
| HL diagnosis during pregnancy | 9 | 26 | 348 | 0.05 | (0.02, 0.10) | 49.06 |
| Never diagnosed with HL | 2 | 233 | 2,034 | 0.11 | (0.10, 0.13) | - |
| ***Stillbirth*** | | | | | | |
| **Studies published prior to 2000 excluded** | | | | | | |
| Treatment for HL before pregnancy | 4 | 15 | 1,752 | 0.01 | (0.00, 0.01) | 0.00 |
| HL diagnosis during pregnancy | 3 | 2 | 193 | 0.00 | (0.00, 0.02) | - |
| Never diagnosed with HL | 2 | 50 | 11,856 | 0.00 | (0.00, 0.01) | - |
| **Studies published prior to 1990 excluded** | | | | | | |
| Treatment for HL before pregnancy | 7 | 17 | 1,843 | 0.00 | (0.00, 0.01) | 0.00 |
| HL diagnosis during pregnancy | 5 | 4 | 242 | 0.00 | (0.00, 0.02) | 0.00 |
| Never diagnosed with HL | 5 | 89,686 | 13,066,788 | 0.00 | (0.00, 0.01) | 78.65 |
| ***Induction of labour*** | | | | | | |
| **Studies published prior to 2000 excluded** | | | | | | |
| Treatment for HL before pregnancy | 2 | 22 | 669 | 0.02 | (0.01, 0.03) | - |
| HL diagnosis during pregnancy | 0 | - | - | - | - | - |
| Never diagnosed with HL | 1 | 97,054 | 7,916,388 | 0.01 | (0.01, 0.01) | - |
| **Studies published prior to 1990 excluded** | | | | | | |
| Treatment for HL before pregnancy | 3 | 22 | 678 | 0.08 | (0.00, 0.43) | - |
| HL diagnosis during pregnancy | 0 | - | - | - | - | - |
| Never diagnosed with HL | 1 | 97,054 | 7,916,388 | 0.01 | (0.01, 0.01) | - |

**Appendix H.** Risk of bias assessment

**Table S22.** Level of bias in studies of patients diagnosed with and treated for Hodgkin lymphoma before pregnancy.

| **Study** | **Selection bias** | **Exposure bias** | **Outcome bias** | **Confounding** | **Analytic bias** | **Attrition bias** | **Overall risk of bias** |
| --- | --- | --- | --- | --- | --- | --- | --- |
| Anderson et al., 2017 | Low: Sample selected from North Carolina Cancer Registry and rationale for case and control selection explained. | Low: Assessment of exposure from Central Cancer Registry records. | Low: Assessment of outcome from Central Cancer Registry records linked to statewide birth certificate files. | Low: Certain confounders assessed: year of birth, maternal age, race/ethnicity, maternal education, previous live births, marital status, maternal smoking during pregnancy. | Low: Sample size calculation not performed, but all available eligible patients studied. | Low: < 10% loss to follow up and reasons not explained. | Low |
| Hartnett et al., 2017 | Low: Sample selected from cancer registries in the states of Georgia, North Carolina and Tennessee and rationale for case and control selection explained. | Low: Assessment of exposure from cancer registry and birth certificate data linked to vital records. | Low: Assessment of outcome from cancer registry and birth certificate data linked to vital records. | Low: Certain confounders assessed: maternal age at delivery, race, ethnicity, parity, maternal education, smoking status during pregnancy, marital status. | Low: Sample size calculation not performed, but all available eligible patients studied. | Minimal: All subjects from initiation of study to final outcomes assessment accounted for. | Low |
| Reulen et al., 2017 | Low: Sample selected from British Childhood Cancer Survivor Study, of which the cohort was ascertained through the National Registry of Childhood Tumours (NRCT). | Low: Assessment of exposure from Childhood Cancer Research Group. | Low: Assessment of outcome from Hospital Episode Statistics for England. | Low: Certain confounders assessed: maternal age, parity. | Low: Sample size calculation not performed, but all available eligible patients studied. | Low: < 10% loss to follow up and reasons not explained. | Low |
| De Sanctis et al., 2012 | Low: Sample selected from all women with HL treated at Department of Hematology and Radiotherapy, University "Sapienza" of Rome, 1972-1999. | Minimal: Assessment of exposure from hospital records. | Minimal: Assessment of outcome from hospital records. | Low: Certain confounders assessed: maternal age at diagnosis and at each gestational event, hormonal therapies during and/or after treatment and relapse or progression occurrence. | Low: Sample size calculation not performed, but all available eligible patients studied. | Minimal: All subjects from initiation of study to final outcomes assessment accounted for. | Low |
| Reulen et al., 2009 | Low: Sample selected from British Childhood Cancer Survivor Study, of which the cohort was ascertained through the National Registry of Childhood Tumours (NRCT). | Low: Assessment of exposure from Childhood Cancer Research Group. | Low: Assessment of outcome from postal patient questionnaires. | Low: Certain confounders assessed: maternal age, birth order, exposure to chemotherapy and radiotherapy. Pregnancy termination outcome was additionally adjusted for decade of treatment. | Low: Sample size calculation not performed, but all available eligible patients studied. | Low: < 10% loss to follow up and reasons not explained. | Low |
| Langagergaard et al., 2008 | Minimal: Sample selected from large Danish population registries and rationale for case and control selection explained. | Low: Assessment of exposure from Danish Cancer Registry | Low: Assessment of outcome from Danish Birth and National Hospital Discharge Registry. | Low: Certain confounders assessed: maternal age, parity, calendar period of birth, gender of newborn, treatment with radiotherapy, gestational age. | Low: Sample size calculation not performed, but all available eligible patients studied. | Low: < 10% loss to follow up and reasons not explained. | Low |
| Signorello et al., 2006 | Low: Sample selected from Childhood Cancer Survivor Study with sibling controls. | Low: Assessment of exposure from postal patient questionnaires. | Low: Assessment of outcome from postal patient questionnaires. | Low: Certain confounders assessed: maternal age, birth order, sex of the child, maternal alcohol drinking, maternal smoking, and use of assisted reproductive technology | Low: Sample size calculation not performed, but all available eligible patients studied. | Low: < 10% loss to follow up and reasons not explained. | Low |
| Roman et al., 2005 | Low: Sample selected from UK Childhood Cancer Study with rationale for case and control selection explained. | Minimal: Assessment of exposure from hospital records. | Minimal: Assessment of outcome from hospital records. | Low: Certain confounders assessed: sex of child, age at diagnosis, UKCCS study region. | Low: Sample size calculation not performed, but all available eligible patients studied. | Low: < 10% loss to follow up and reasons not explained. | Low |
| Green et al., 2002 | Low: Sample selected from Childhood Cancer Survivor Study with sibling controls. | Minimal: Assessment of exposure from hospital records. | Low: Assessment of outcome from hospital records and patient questionnaires. | Low: Certain confounders assessed: maternal age, live birth order, maternal tobacco use during pregnancy, maternal alcohol use, maternal education, pelvic irradiation, treatment with an alkylating agent, with non-alkylating agent chemotherapy, with actinomycin D, with daunorubicin or doxorubicin, with any chemotherapy other than an alkylating agent, actinomycin D, daunorubicin or doxorubicin. | Low: Sample size calculation not performed, but all available eligible patients studied. | Low: < 10% loss to follow up and reasons not explained. | Low |
| Brierley et al., 1998 | Low: Sample selected from Princess Margaret Hospital database and rationale for case and control selection explained. | Low: Assessment of exposure from postal patient questionnaires. | Low: Assessment of outcome from postal patient questionnaires. | Moderate: Not assessed for confounders | Low: Sample size calculation not performed, but all available eligible patients studied. | High: > 20% did not complete questionnaire regarding fertility. | Low-moderate |
| Swerdlow et al., 1996 | Low: Sample selected from patients with HL treated at Mount Vernon Hospital, Middlesex, England with rationale for case and control selection explained. | Minimal: Assessment of exposure from hospital records. | Minimal: Assessment of exposure from hospital records, postal questionnaires and interviews. | Moderate: Not assessed for confounders | Low: Sample size calculation not performed, but all available eligible patients studied. | Low: < 10% loss to follow up and reasons not explained. | Low-moderate |
| Aisner et al., 1993 | Low: Sample selected from all patients with HL treated at the University of Maryland Cancer Center, Baltimore, United States, 1965-1985. | Low: Assessment of exposure from interviews and patient questionnaires. | Low: Assessment of outcome from interviews and patient questionnaires. | Moderate: Not assessed for confounders | Low: Sample size calculation not performed, but all available eligible patients studied. | Low: < 10% loss to follow up and reasons not explained. | Low-moderate |
| Janov et al., 1992 | Low: Sample selected from Cancer and Leukemia Group B research group. | Minimal: Assessment of exposure from telephone questionnaires. | Low: Assessment of outcome from telephone questionnaires. | Moderate: Not assessed for confounders | Low: Sample size calculation not performed, but all available eligible patients studied. | Low: < 10% loss to follow up and reasons not explained. | Low-moderate |
| [Balcewicz-Sablińska , 1990](https://pubmed.ncbi.nlm.nih.gov/?size=200&term=Balcewicz-Sabli%C5%84ska+K&cauthor_id=2260411) | Low: Sample selected from all pregnant women treated at Institute of Haematology and Blood Transfusion, Warsaw, Poland. | Minimal: Assessment of exposure from hospital records | Minimal: Assessment of outcome from hospital records with some data on childbirth and development obtained via questionnaires. | Moderate: Not assessed for confounders | Low: Sample size calculation not performed, but all available eligible patients studied. | Minimal: All subjects accounted for. | Low-moderate |
| Lacher et al., 1986 | Low: Sample selected from all patients with HL treated at Memorial Sloan-Kettering Cancer Center, New York, United States, August 1973-November 1981. | Minimal: Assessment of exposure from hospital records, patient questionnaires, and direct personal patient communication | Low: Assessment of exposure from hospital records, patient questionnaires, and direct personal patient communication | Moderate: Not assessed for confounders | Low: Sample size calculation not performed, but all available eligible patients studied. | Minimal: All subjects from initiation of study to final outcomes assessment accounted for. | Low-moderate |
| Slanina et al., 1985 | Low: Sample selected from all patients with HL treated at Freiburg im Breisgau, Germany, 1949-1981. | Low: Assessment of exposure from hospital records and patient questionnaires. | Low: Assessment of exposure from hospital records and patient questionnaires. | Moderate: Not assessed for confounders | Low: Sample size calculation not performed, but all available eligible patients studied. | Low: < 10% loss to follow up and reasons not explained. | Low-moderate |
| McKeen et al., 1979 | Low: Sample selected from Cancer and Leukemia Group B research group. | Minimal: Assessment of exposure from direct questioning to participants of Cancer and Leukemia Group B. | Low: Assessment of outcome from direct questioning of living patients and their physicians. | Moderate: Not assessed for confounders | Low: Sample size calculation not performed, but all available eligible patients studied. | Minimal: All subjects from initiation of study to final outcomes assessment accounted for. | Low-moderate |
| Holmes et al., 1978 | Low: Sample selected from University of Kansas Medical Center, College of Health Sciences and Hospital database and rationale for case and control selection explained. | Minimal: Assessment of exposure from hospital records. | Minimal: Assessment of outcome from hospital records. | Moderate: Not assessed for confounders | Moderate: Sample size calculation not performed, unclear if all eligible patients studied. | Minimal: All subjects from initiation of study to final outcomes assessment accounted for. | Low-moderate |
| Le Floch et al., 1976 | Low: Sample selected from all patients with HL who underwent oophoropexy followed by total nodal irradiation at Stanford University Medical Center, California, United States, 1968-1976 | Minimal: Assessment of exposure from hospital records. | Minimal: Assessment of outcome from hospital records. | Moderate: Not assessed for confounders | Low: Sample size calculation not performed, but all available eligible patients studied. | Minimal: All subjects from initiation of study to final outcomes assessment accounted for. | Low-moderate |

**Table S23.** Level of bias in studies of patients with Hodgkin lymphoma during pregnancy.

| **Study** | **Selection bias** | **Exposure bias** | **Outcome bias** | **Confounding** | **Analytic bias** | **Attrition bias** | **Overall risk of bias** |
| --- | --- | --- | --- | --- | --- | --- | --- |
| Kroll-Balcerzak et al., 2019 | Moderate: Sample selection ambiguous but sample may be representative, unclear if all eligible patients included. | High: Method of assessment of exposure not defined. | High: Method of assessment of outcome not defined. | Moderate: Not assessed for confounders | Moderate: Sample size calculation not performed, unclear if all eligible patients studied. | Minimal: All subjects from initiation of study to final outcomes assessment accounted for. | Moderate |
| Maggen et al., 2019 | Minimal: Sample selected from the International Network on Cancer, Infertility and Pregnancy (INCIP) registry. Eligibility criteria and rationale for case and control selection explained. | Low: Assessment of exposure from INCIP registry | Low: Assessment of exposure from INCIP registry | Low: Controls matched for stage and prognostic score at diagnosis. | Low: Sample size calculation not performed, but all available eligible patients studied. | Low: < 10% loss to follow up and reasons not explained. | Low |
| Niu et al., 2019 | Minimal: Sample selected from large population registry and rationale for case and control selection explained. | Low: Assessment of exposure from Washington State-linked vital-hospital discharge records. | Low: Assessment of exposure from Washington State-linked vital-hospital discharge records. | Low: Certain confounders assessed: delivery year, maternal age, marital status, insurance, parity. | Low: Sample size calculation not performed, but all available eligible patients studied. | Low: < 10% loss to follow up and reasons not explained. | Low |
| Avilés et al., 2018 | Low: Sample selected from all pregnant women treated at Oncology Hospital National Medical Center, IMSS in Mexico, 1988-2013. | Minimal: Assessment of exposure from hospital records | Minimal: Assessment of outcome from hospital records | Moderate: Not assessed for confounders | Low: Sample size calculation not performed, but all available eligible patients studied. | Minimal: All subjects from initiation of study to final outcomes assessment accounted for. | Low-moderate |
| Pinnix et al., 2016 | Low: Sample selected from all pregnant women treated at The University of Texas MD Anderson Cancer Center, Texas, United States, 1991-2014. | Minimal: Assessment of exposure from hospital records | Minimal: Assessment of outcome from hospital records | Moderate: Not assessed for confounders | Low: Sample size calculation not performed, but all available eligible patients studied. | Low: < 10% loss to follow up and reasons not explained. | Low-moderate |
| El-Messidi et al., 2015 | Minimal: Sample selected from general population rather than a select group. | Minimal: Assessment of exposure from hospital records | Minimal: Assessment of outcome from hospital records | Low: Certain confounders assessed: baseline characteristics. | Low: Sample size calculation not performed, but all available eligible patients studied. | Minimal: All subjects from initiation of study to final outcomes assessment accounted for. | Low |
| Van Calsteren, 2015 | High: Sample likely not representative, treating physicians requested to register patients. | Minimal: Assessment of exposure from hospital records | Minimal: Assessment of outcome from hospital records. | Low: Certain confounders assessed: gestational age, sex | Low: Sample size calculation not performed, but all available eligible patients studied. | Low: < 10% loss to follow up and reasons not explained. | High |
| Evens et al., 2013 | Low: Sample selected from all pregnant women treated at 11 academic centres in the United States, 1999-2011. | Minimal: Assessment of exposure from hospital records | Minimal: Assessment of outcome from hospital records | Moderate: Not assessed for confounders | Low: Sample size calculation not performed, but all available eligible patients studied. | Low: < 10% loss to follow up and reasons not explained. | Low-moderate |
| Langagergaard et al., 2008 | Minimal: Sample selected from large Danish population registries and rationale for case and control selection explained. | Low: Assessment of exposure from Danish Cancer Registry | Low: Assessment of outcome from Danish Birth and National Hospital Discharge Registry. | Low: Certain confounders assessed: maternal age, parity, calendar period of birth, gender of newborn, treatment with radiotherapy, gestational age. | Low: Sample size calculation not performed, but all available eligible patients studied. | Low: < 10% loss to follow up and reasons not explained. | Low |
| Dilek et al. 2006 | Moderate: Sample selection ambiguous but sample may be representative, unclear if all eligible patients included. | Minimal: Assessment of exposure from hospital records | Minimal: Assessment of outcome from hospital records. | Moderate: Not assessed for confounders | Moderate: Sample size calculation not performed, unclear if all eligible patients studied. | Minimal: All subjects from initiation of study to final outcomes assessment accounted for. | Moderate |
| Lishner et al., 1992 | Low: Sample selected from Princess Margaret Hospital database and rationale for case and control selection explained. | Minimal: Assessment of exposure from hospital records | Minimal: Assessment of outcome from hospital records | Low: Controls matched for stage and prognostic score at diagnosis. | Low: Sample size calculation not performed, but all available eligible patients studied. | Low: < 10% loss to follow up and reasons not explained. | Low |
| Woo et al., 1992 | Low: Sample selected from all pregnant women treated at The University of Texas MD Anderson Cancer Center, Texas, United States, 1956-1990. | Minimal: Assessment of exposure from hospital records | Minimal: Assessment of outcome from hospital records with some data obtained via telephone questionnaire. | Moderate: Not assessed for confounders | Low: Sample size calculation not performed, but all available eligible patients studied. | Low: < 10% loss to follow up and reasons not explained. | Low-moderate |
| Balcewicz-Sablińska , 1990 | Low: Sample selected from all pregnant women treated at Institute of Haematology and Blood Transfusion, Warsaw, Poland | Minimal: Assessment of exposure from hospital records | Minimal: Assessment of outcome from hospital records with some data on childbirth and development obtained via questionnaire. | Moderate: Not assessed for confounders | Low: Sample size calculation not performed, but all available eligible patients studied. | Minimal: All subjects accounted for. | Low-moderate |
| Nisce et al., 1986 | Low: Sample selected from all pregnant women treated at Memorial Sloan-Kettering Cancer Center, New York, United States, 1969-1982. | Minimal: Assessment of exposure from hospital records | Minimal: Assessment of outcome from hospital records. | Moderate: Not assessed for confounders | Low: Sample size calculation not performed, but all available eligible patients studied. | Minimal: All subjects from initiation of study to final outcomes assessment accounted for. | Low-moderate |
| Tawil et al., 1985 | Low: Sample selected from all pregnant women treated at Hôpital Maisonneuve-Rosemont, Montreal, Canada, 1968-1979. | Minimal: Assessment of exposure from hospital records | Minimal: Assessment of outcome from hospital records. | Moderate: Not assessed for confounders | Low: Sample size calculation not performed, but all available eligible patients studied. | Minimal: All subjects from initiation of study to final outcomes assessment accounted for. | Low-moderate |
| Jacobs et al., 1981 | Low: Sample selected from all pregnant women treated at Stanford University Medical Center, California, United States, 1963-1979. | Minimal: Assessment of exposure from hospital records | Minimal: Assessment of outcome from hospital records. | Moderate: Not assessed for confounders | Low: Sample size calculation not performed, but all available eligible patients studied. | Minimal: All subjects from initiation of study to final outcomes assessment accounted for. | Low-moderate |
| McKeen et al., 1979 | Low: Sample selected from Cancer and Leukemia Group B research group. | Minimal: Assessment of exposure from direct questioning to participants of Cancer and Leukemia Group B. | Low: Assessment of outcome from direct questioning of living patients and their physicians. | Moderate: Not assessed for confounders | Low: Sample size calculation not performed, but all available eligible patients studied. | Minimal: All subjects from initiation of study to final outcomes assessment accounted for. | Low-moderate |

**Appendix I.** Certainty of evidence assessed using the GRADE approach for each outcome.

| **Outcome measure** | **Number of studies** | **Risk of bias** | **Inconsistency** | **Indirectness** | **Imprecision** | **Publication bias** | **Overall certainty of evidence** |
| --- | --- | --- | --- | --- | --- | --- | --- |
| Congenital malformations | 20 | Serious^a^ | Not serious^b^ | Not serious | Serious^d^ | Inconclusive | Low |
| Preterm birth | 21 | Serious^a^ | Not serious^b^ | Not serious | Serious^d^ | Inconclusive | Low |
| Miscarriage | 23 | Serious^a^ | Not serious^b^ | Not serious | Serious^d^ | Inconclusive | Low |
| Premature rupture of membranes | 4 | Serious^a^ | Not serious^b^ | Not serious | Serious^c^ | Inconclusive | Very low |
| Postpartum haemorrhage | 3 | Serious^a^ | Not serious^b^ | Not serious | Serious^c^ | Inconclusive | Very low |
| Anaemia | 4 | Serious^a^ | Not serious^b^ | Not serious | Serious^c^ | Inconclusive | Very low |
| 5-minute APGAR score < 7 | 4 | Serious^a^ | Not serious^b^ | Not serious | Serious^c^ | Inconclusive | Very low |
| Low birth weight | 16 | Serious^a^ | Not serious^b^ | Not serious | Serious^d^ | Inconclusive | Low |
| Small for gestational age | 9 | Serious^a^ | Not serious^b^ | Not serious | Serious^d^ | Inconclusive | Low |
| Neonatal death | 6 | Serious^a^ | Not serious^b^ | Not serious | Serious^c^ | Inconclusive | Very low |
| Caesarean section | 11 | Serious^a^ | Not serious^b^ | Not serious | Serious^d^ | Inconclusive | Low |
| Pregnancy-induced hypertension | 2 | Serious^a^ | Not serious^b^ | Not serious | Serious^c^ | Inconclusive | Very low |
| Pre-eclampsia | 5 | Serious^a^ | Not serious^b^ | Not serious | Serious^c^ | Inconclusive | Very low |
| Gestational diabetes | 4 | Serious^a^ | Not serious^b^ | Not serious | Serious^c^ | Inconclusive | Very low |
| Elective termination of pregnancy | 22 | Serious^a^ | Not serious^b^ | Not serious | Serious^d^ | Inconclusive | Low |
| Stillbirth | 14 | Serious^a^ | Not serious^b^ | Not serious | Serious^d^ | Inconclusive | Low |
| Induction of labour | 5 | Serious^a^ | Not serious^b^ | Not serious | Serious^c^ | Inconclusive | Very low |
| Blood transfusion | 1 | Serious^a^ | N/A | Not serious | Serious^c^ | Inconclusive | Very low |
| Venous thromboembolism | 1 | Serious^a^ | N/A | Not serious | Serious^c^ | Inconclusive | Very low |
| Chorioamnionitis | 1 | Serious^a^ | N/A | Not serious | Serious^c^ | Inconclusive | Very low |

a. Serious risk of bias as observational studies, few studies reported adjusted effect estimates and serious risk of confounding.

b. Although substantial to considerable heterogeneity between studies in each group, majority of 95% confidence intervals (CI) overlapped indicating consistency in associations.

c. Effect estimate came from small number of studies.

d. Included studies had wide 95% CI.
